# Supplementary material for: Nanoarchitectonics for Biodegradable Superabsorbent Based on Carboxymethyl Starch and Chitosan Cross-Linked with Vanillin
Source: Int J Mol Sci. 2022 May 11;23(10):5386. doi: 10.3390/ijms23105386 (PMC9142128; doi:10.3390/ijms23105386)
Supplement: Supplementary file 1 [file ijms-23-05386-s001.zip › ijms-1692363-supplementary.pdf]

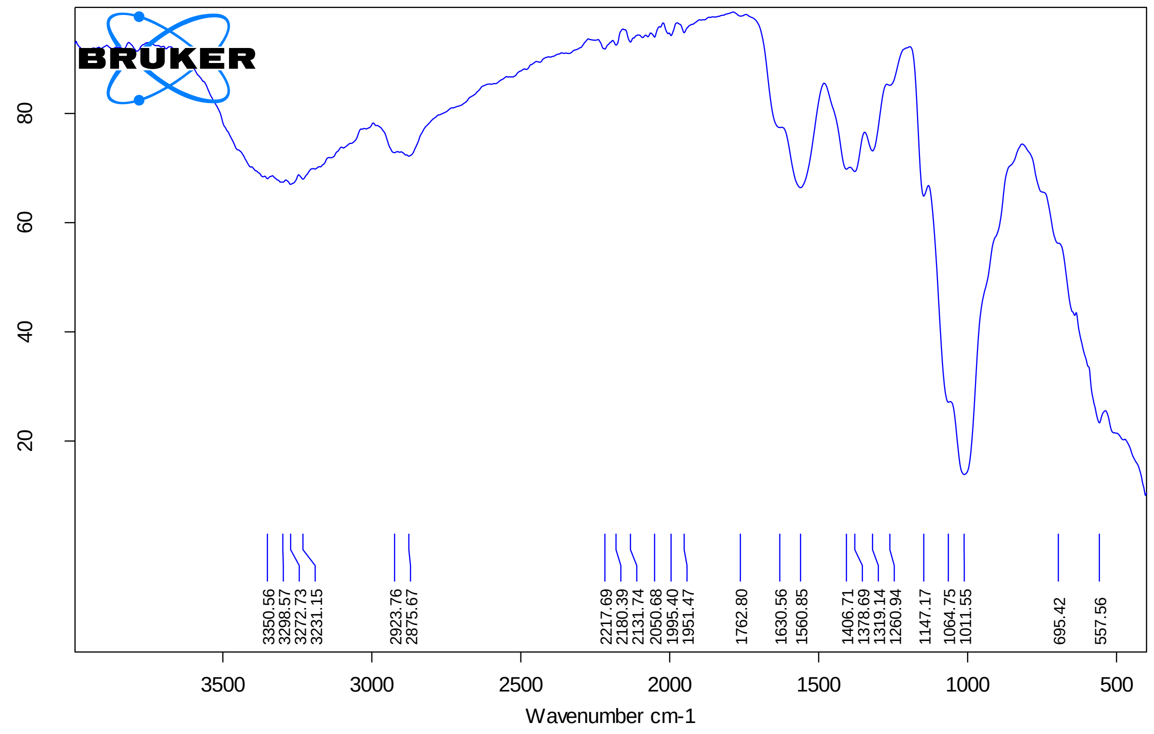

C:\Users\Lenovo\Desktop\Obliczenia\IR syntezy SAP\SAP Natural\CMS-g-Ch\IR pick picking\01.07 1-1-0,01 norm.SPA.0	**01.07. 1-1-0,01	01.09.2020

Page 1/1

Figure S1. FTIR spectrum of the sample prepared with the ratio 1.0 carboxymethyl starch (CMS): 1.0 chitosan (Ch): 0.01 vanillin (Van).

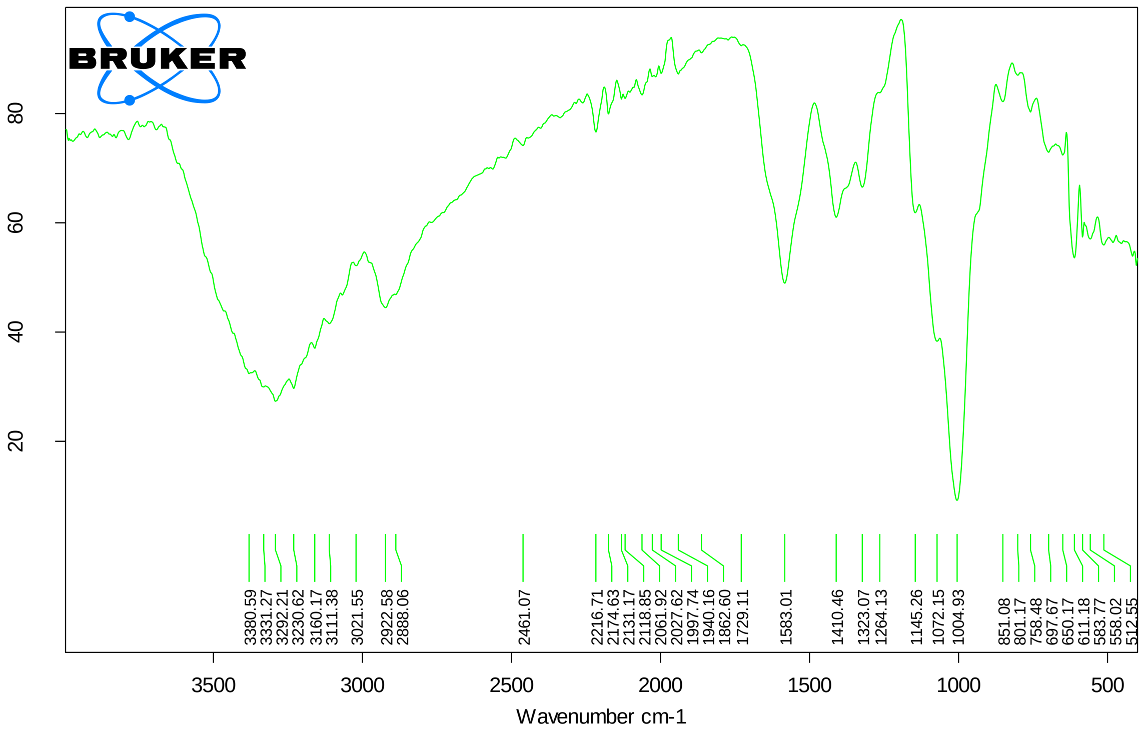

C:\Users\Lenovo\Desktop\Obliczenia\IR syntezy SAP\SAP Natural\CMS-g-Ch\IR pick picking\14.07. I 0,25-1,75-0,01 norm.SPA.0	**14.07. I 0,	01.09.2020

Figure S2. FTIR spectrum of the sample prepared with the ratio 0.25 carboxymethyl starch (CMS): 1.75 chitosan (Ch): 0.01 vanillin (Van).

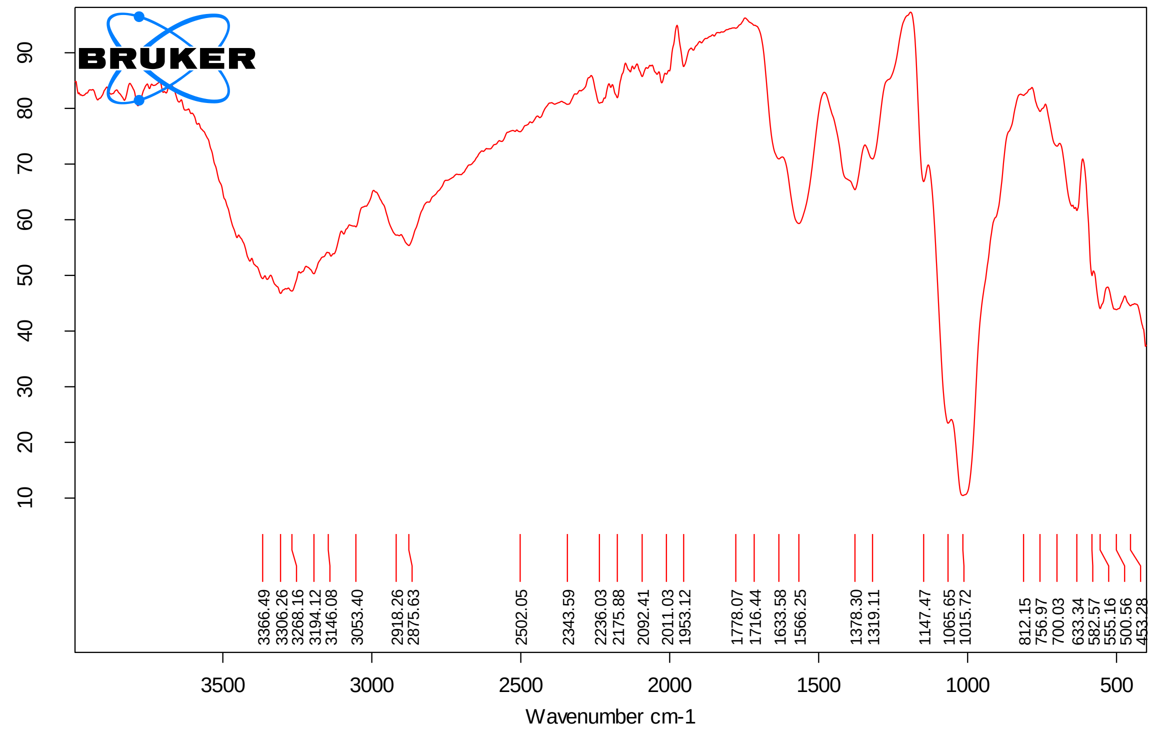

C:\Users\Lenovo\Desktop\Obliczenia\IR syntezy SAP\SAP Natural\CMS-g-Ch\IR pick picking\01.07 1-1-0,02 norm.SPA.0	**01.07. 1-1-0,02	01.09.2020

Page 1/1

Figure S3. FTIR spectrum of the sample prepared with the ratio 1.0 carboxymethyl starch (CMS): 1.0 chitosan (Ch): 0.02 vanillin (Van).

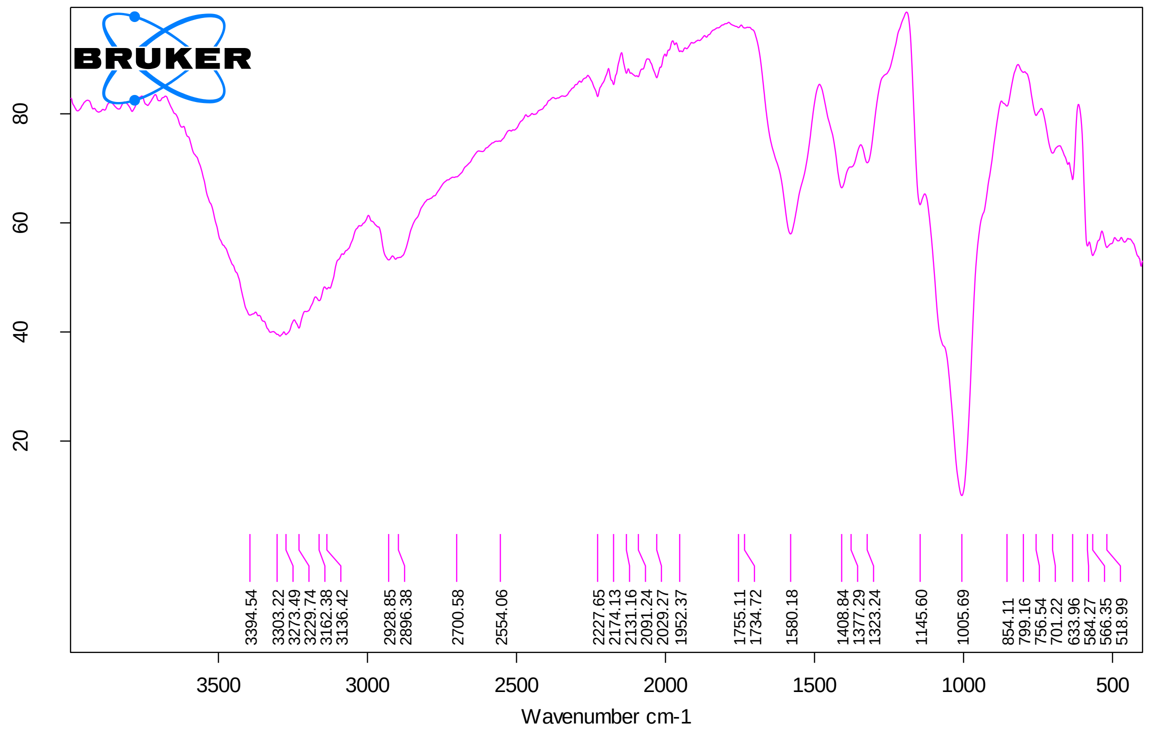

C:\Users\Lenovo\Desktop\Obliczenia\IR syntezy SAP\SAP Natural\CMS-g-Ch\IR pick picking\14.07 0,5-1,5-0,02 norm.SPA.0	**14.07. I 0,5-1,5	01.09.2020

Page 1/1

Figure S4. FTIR spectrum of the sample prepared with the ratio 0.5 carboxymethyl starch (CMS): 1.5 chitosan (Ch): 0.02 vanillin (Van).

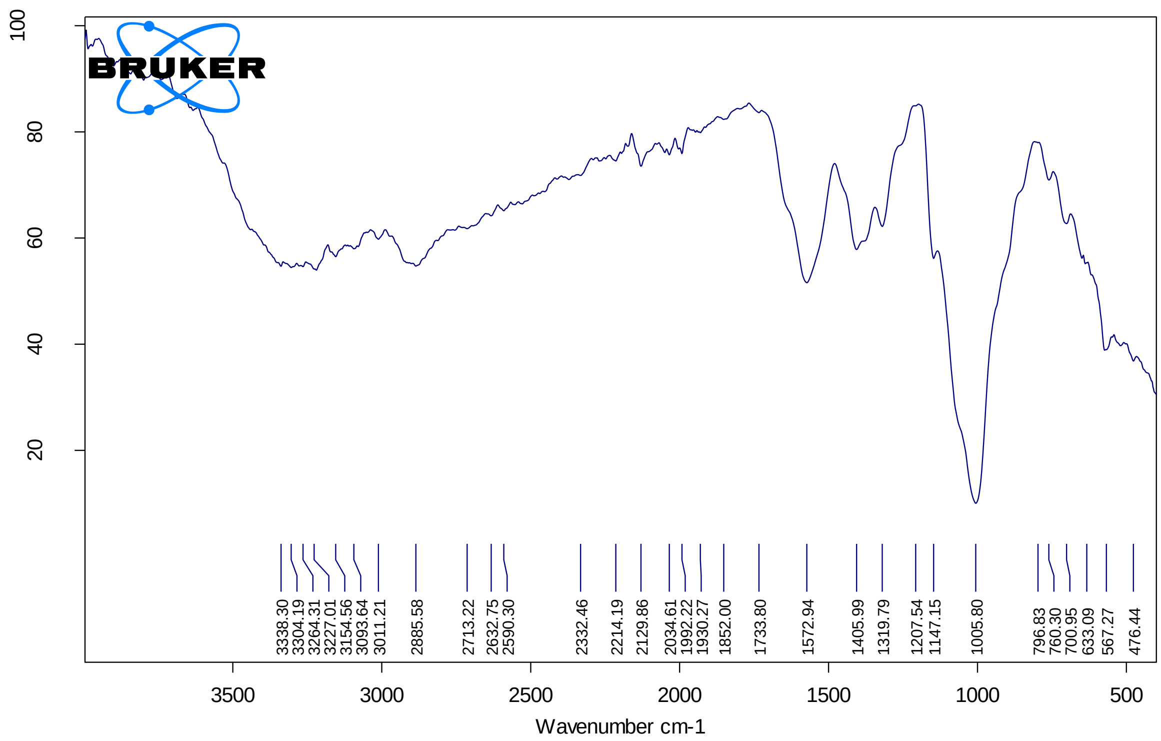

E:\2019\Dokumenty\Doktorat 2018\Obliczenia\IR syntezy SAP\Natural\CMS-g-Ch\publikacja\02.04-0,04.SPA **02.04-0.04	14.05.2020

Page 1/1

Figure S5. FTIR spectrum of the sample prepared with the ratio 1.0 carboxymethyl starch (CMS): 1.0 chitosan (Ch): 0.04 vanillin (Van).

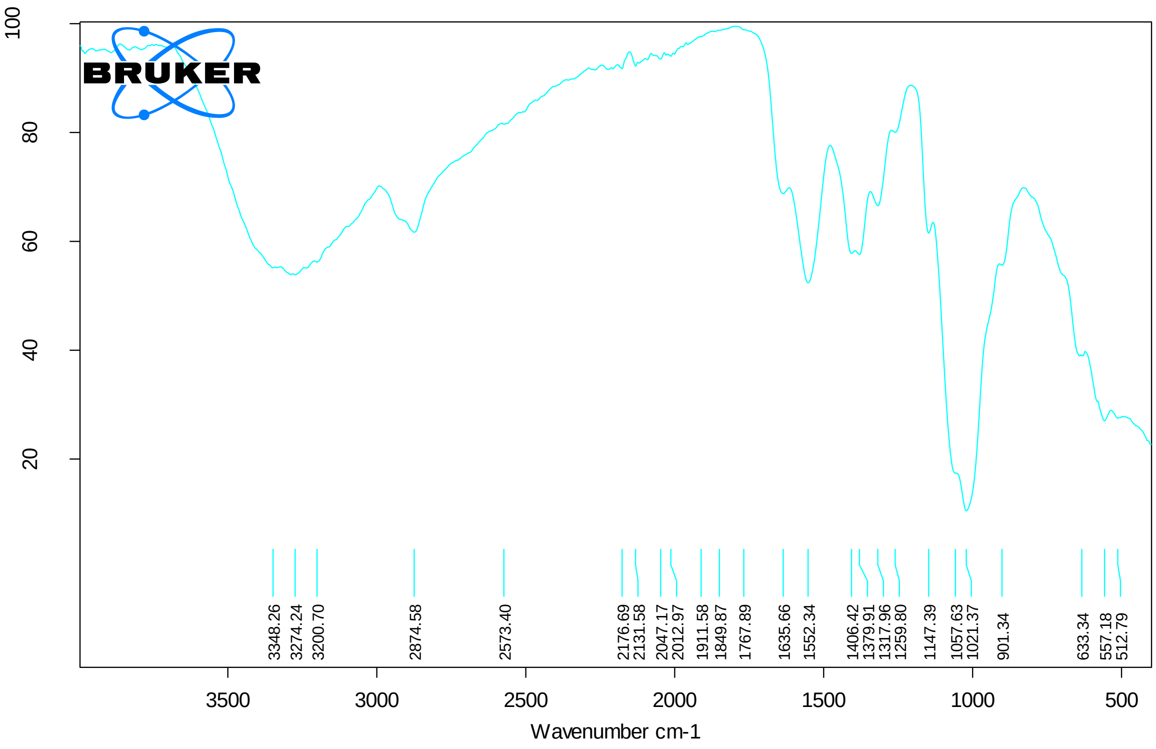

C:\Users\Lenovo\Desktop\Obliczenia\IR syntezy SAP\SAP Natural\CMS-g-Ch\IR pick picking\14.07. III 1,5-0,05-0,06 norm.SPA.0	**14.07. III 1	01.09.2020

Page 1/1

Figure S6. FTIR spectrum of the sample prepared with the ratio 1.5 carboxymethyl starch (CMS): 0.5 chitosan (Ch): 0.06 vanillin (Van).

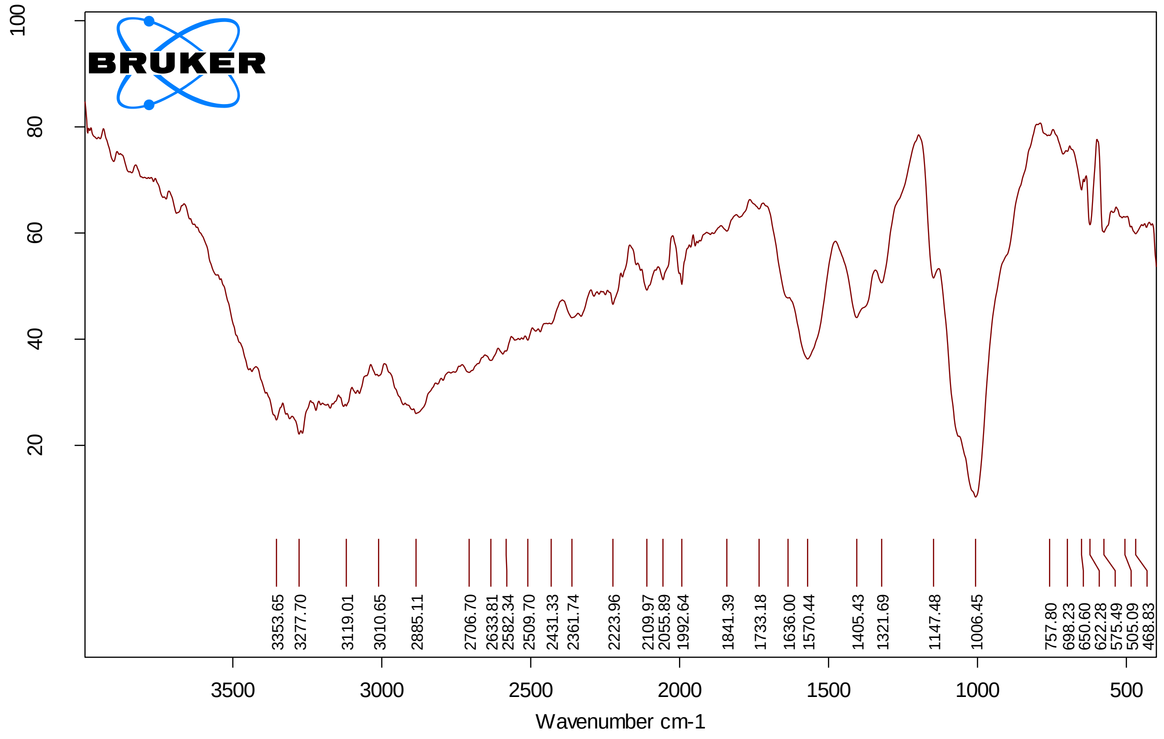

E:\2019\Dokumenty\Doktorat 2018\Obliczenia\IR syntezy SAP\Natural\CMS-g-Ch\publikacja\02.04-0,06.SPA **02.04-0.06	14.05.2020

Page 1/1

Figure S7. FTIR spectrum of the sample prepared with the ratio 1.0 carboxymethyl starch (CMS): 1.0 chitosan (Ch): 0.06 vanillin (Van).

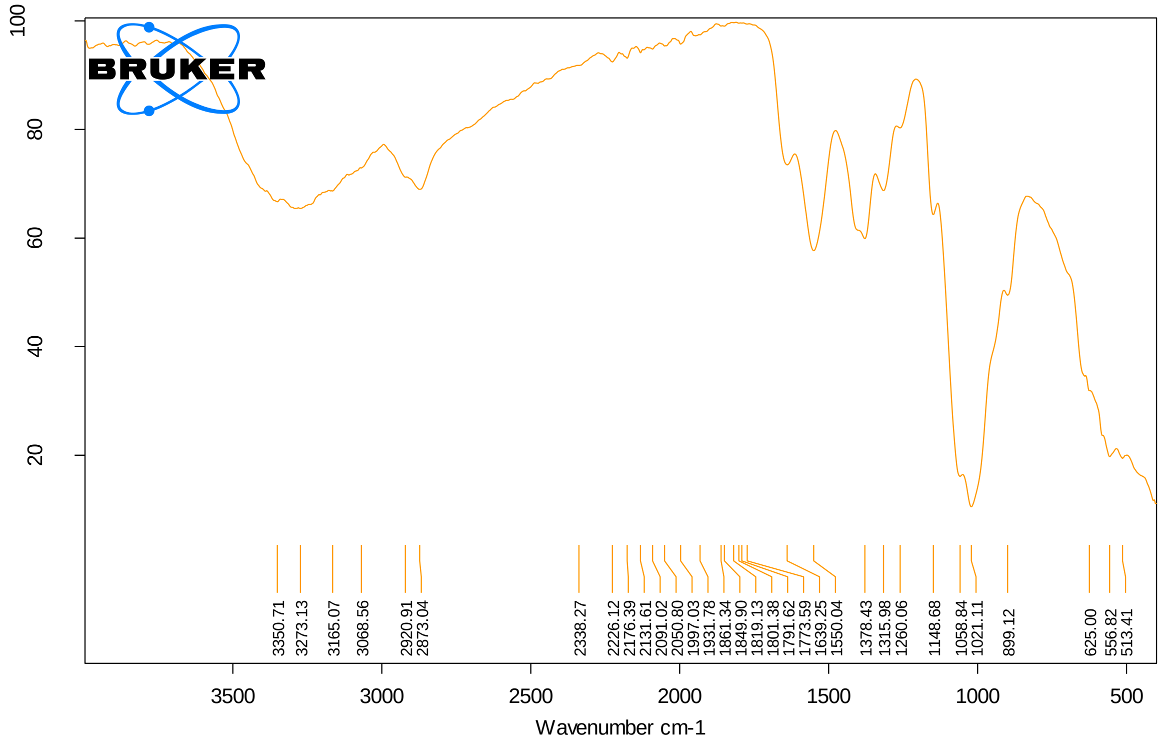

C:\Users\Lenovo\Desktop\Obliczenia\IR syntezy SAP\SAP Natural\CMS-g-Ch\IR pick picking\14.07. IV 1,75-0,25-0,07 norm.SPA.0	**14.07. IV	01.09.2020

Page 1/1

Figure S8. FTIR spectrum of the sample prepared with the ratio 1.75 carboxymethyl starch (CMS): 0.25 chitosan (Ch): 0.07 vanillin (Van).

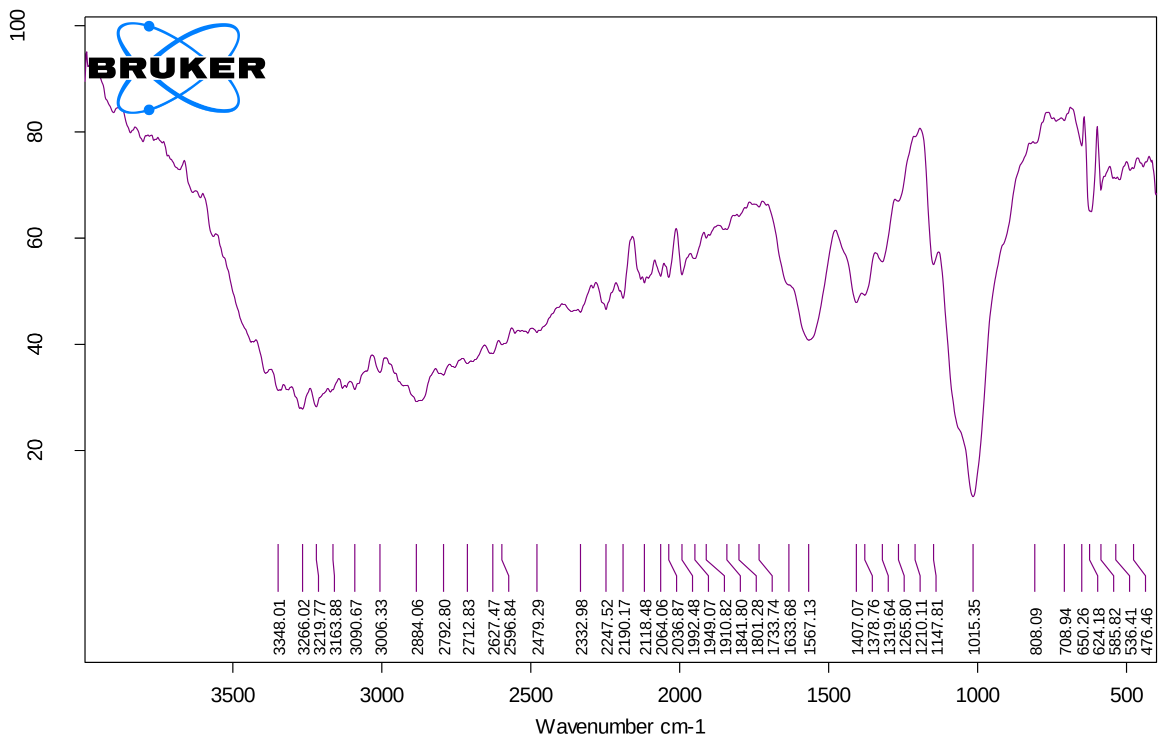

E:\2019\Dokumenty\Doktorat 2018\Obliczenia\IR syntezy SAP\Natural\CMS-g-Ch\publikacja\02.04-0,08.SPA	**02.04-0.08	14.05.2020

Page 1/1

Figure S9. FTIR spectrum of the sample prepared with the ratio 1.0 carboxymethyl starch (CMS): 1.0 chitosan (Ch): 0.08 vanillin (Van).

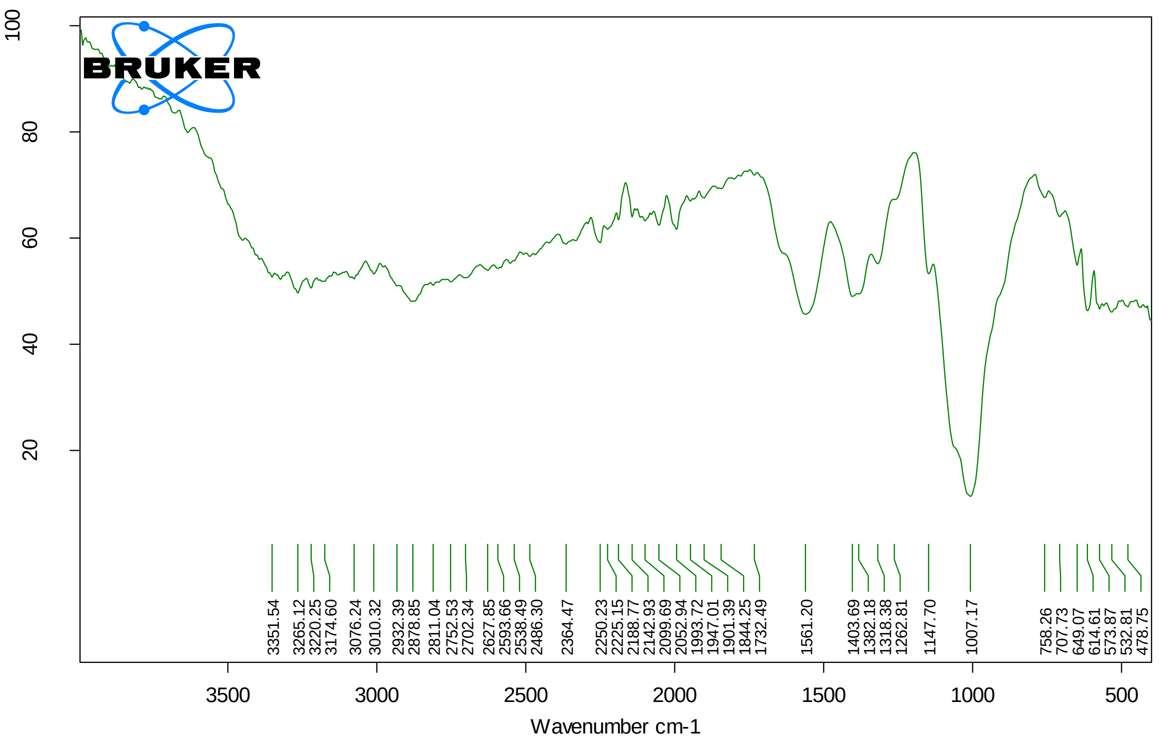

E:\2019\Dokumenty\Doktorat 2018\Obliczenia\IR syntezy SAP\Natural\CMS-g-Ch\publikacja\02.04-0,10.SPA **02.04-0.1	14.05.2020

Page 1/1

Figure S10. FTIR spectrum of the sample prepared with the ratio 1.0 carboxymethyl starch (CMS): 1.0 chitosan (Ch): 0.10 vanillin (Van).

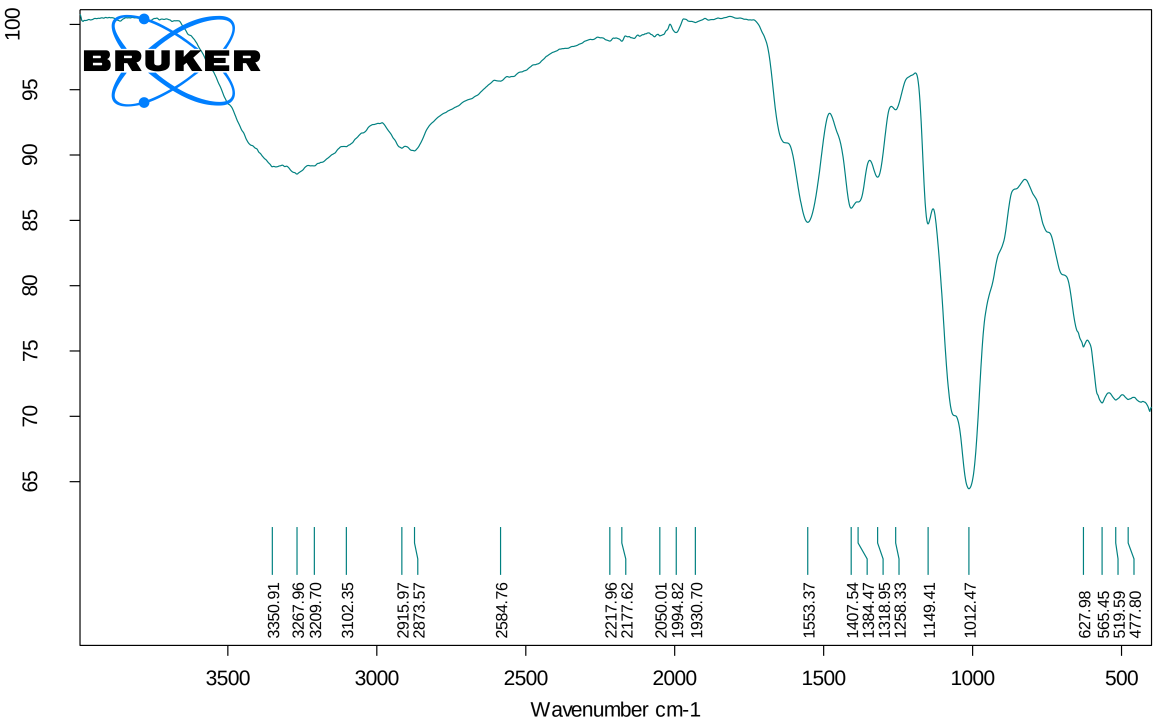

E:\2019\Dokumenty\Doktorat 2018\Obliczenia\IR syntezy SAP\Natural\CMS-g-Ch\publikacja\bez Van.SPA *CMS g--Ch bez Van	05.05.2020

Page 1/1

Figure S11. FTIR spectrum of the sample prepared with the ratio 1.0 carboxymethyl starch (CMS): 1.0 chitosan (Ch).

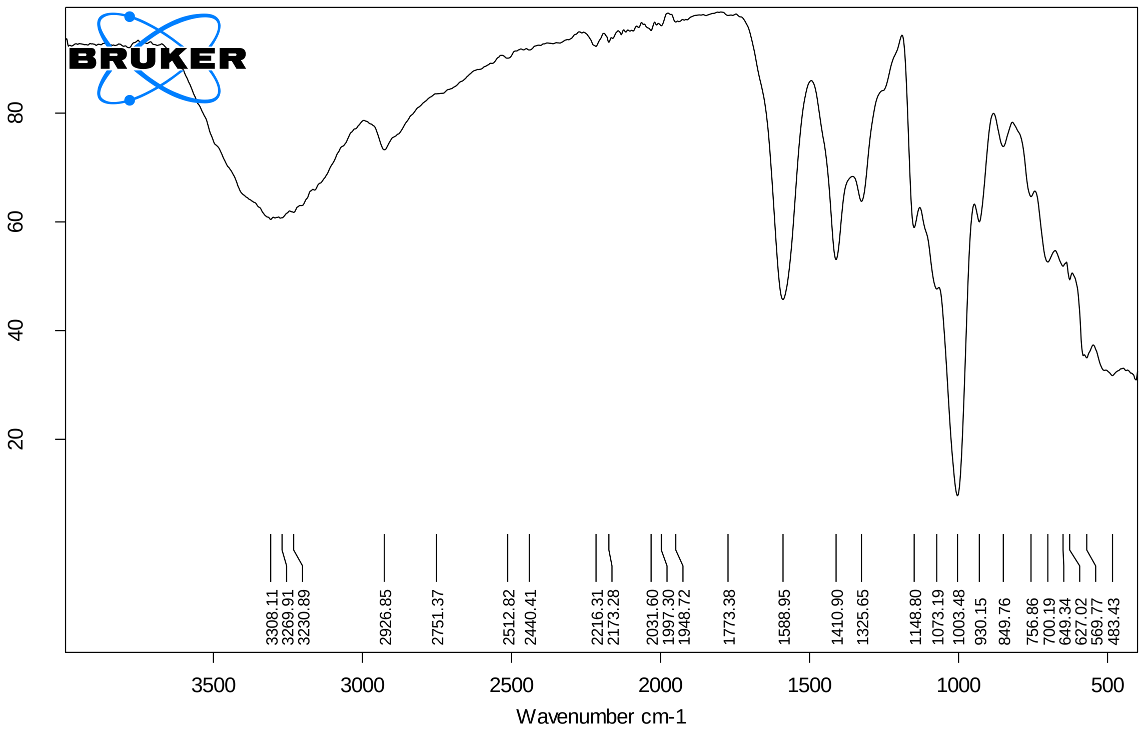

E:\2019\Dokumenty\Doktorat 2018\Obliczenia\IR syntezy SAP\Natural\CMS-g-Ch\publikacja\03.07 CMS.SPA	**03.07. CMS	01.09.2020

Page 1/1

Figure S12. FTIR spectrum of the carboxymethyl starch (CMS).

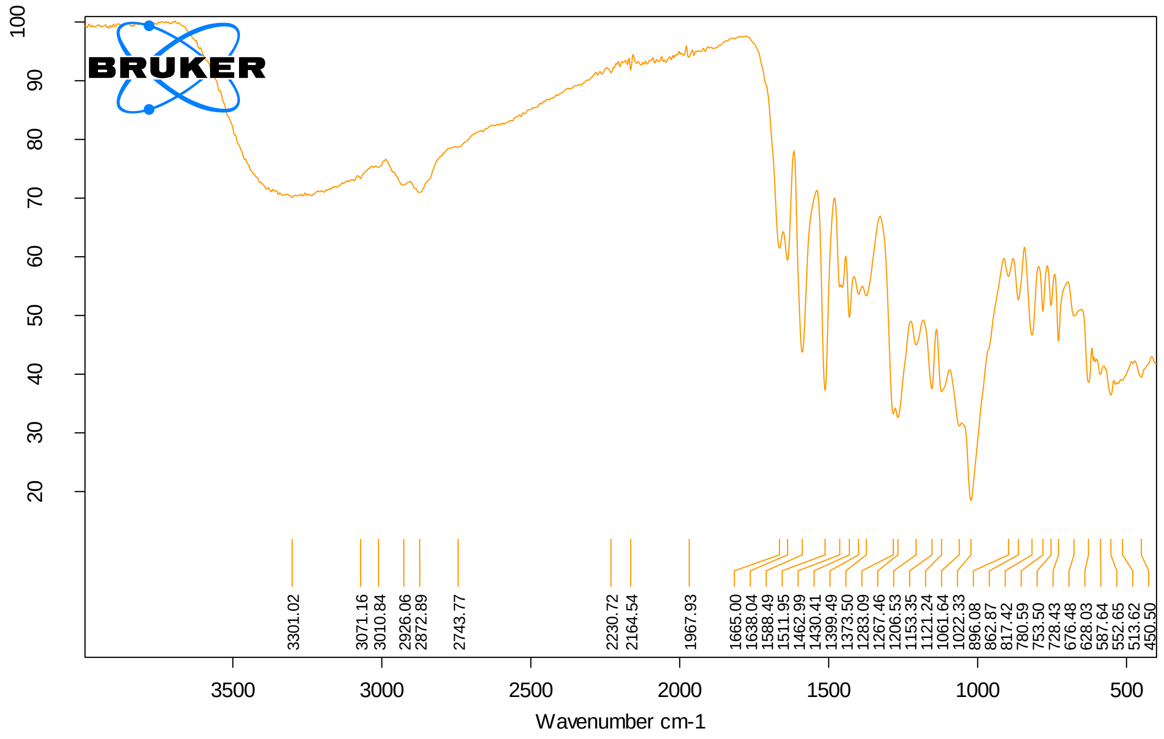

E:\2019\Dokumenty\Doktorat 2018\Obliczenia\IR syntezy SAP\Natural\CMS-g-Ch\publikacja\Ch-V50.SPA	Ch-V50	27.01.2020

Page 1/1

Figure S13. FTIR spectrum of the chitosan (Ch).

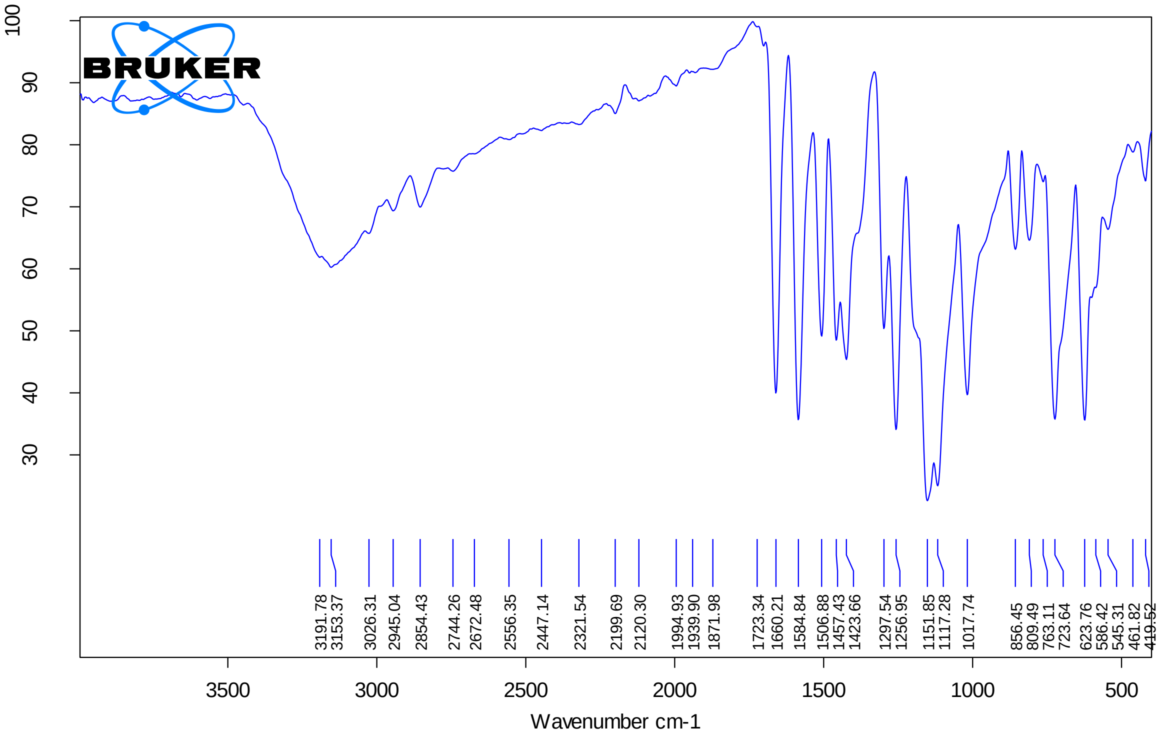

E:\2019\Dokumenty\Doktorat 2018\Obliczenia\IR syntezy SAP\Natural\CMS-g-Ch\publikacja\Van.SPA **Van	28.04.2020

Page 1/1

Figure S14. FTIR spectrum of the vanillin (Van).

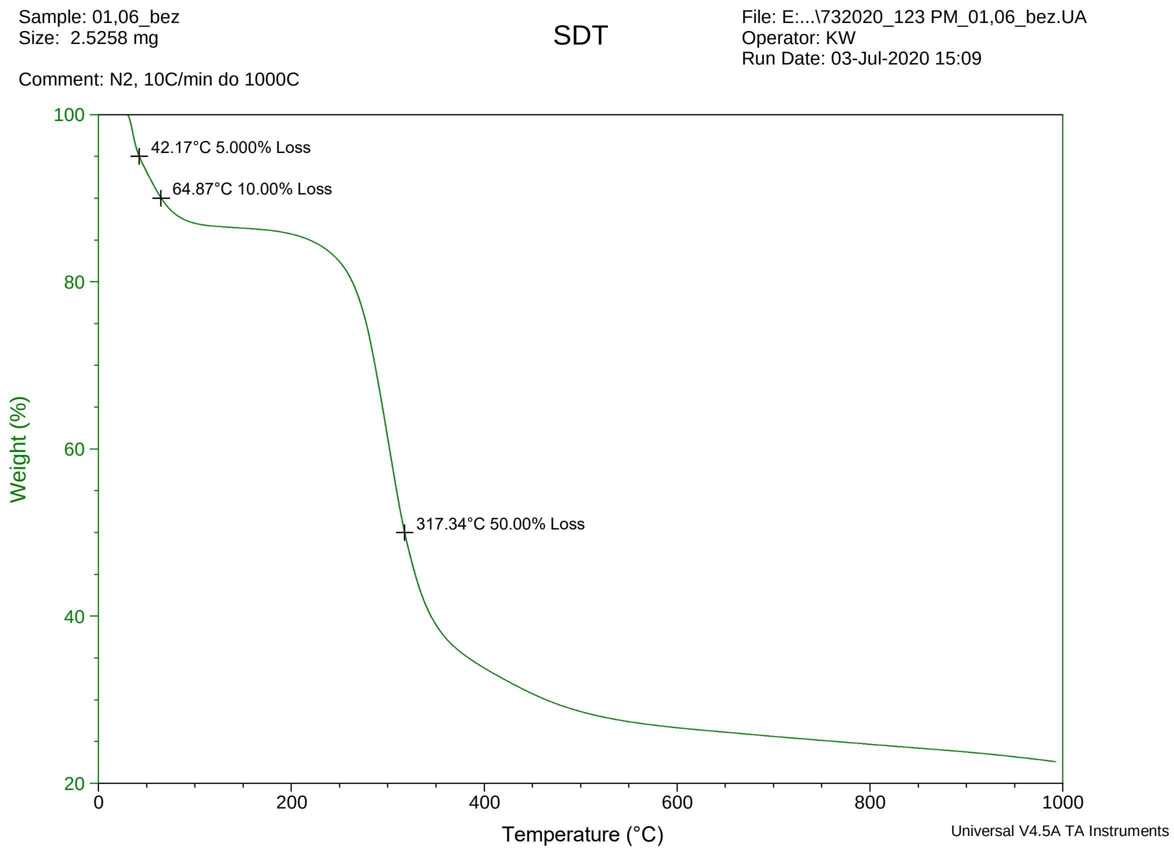

Figure S15. . TGA of 1.0 carboxymethyl starch : 1.0 chitosan.

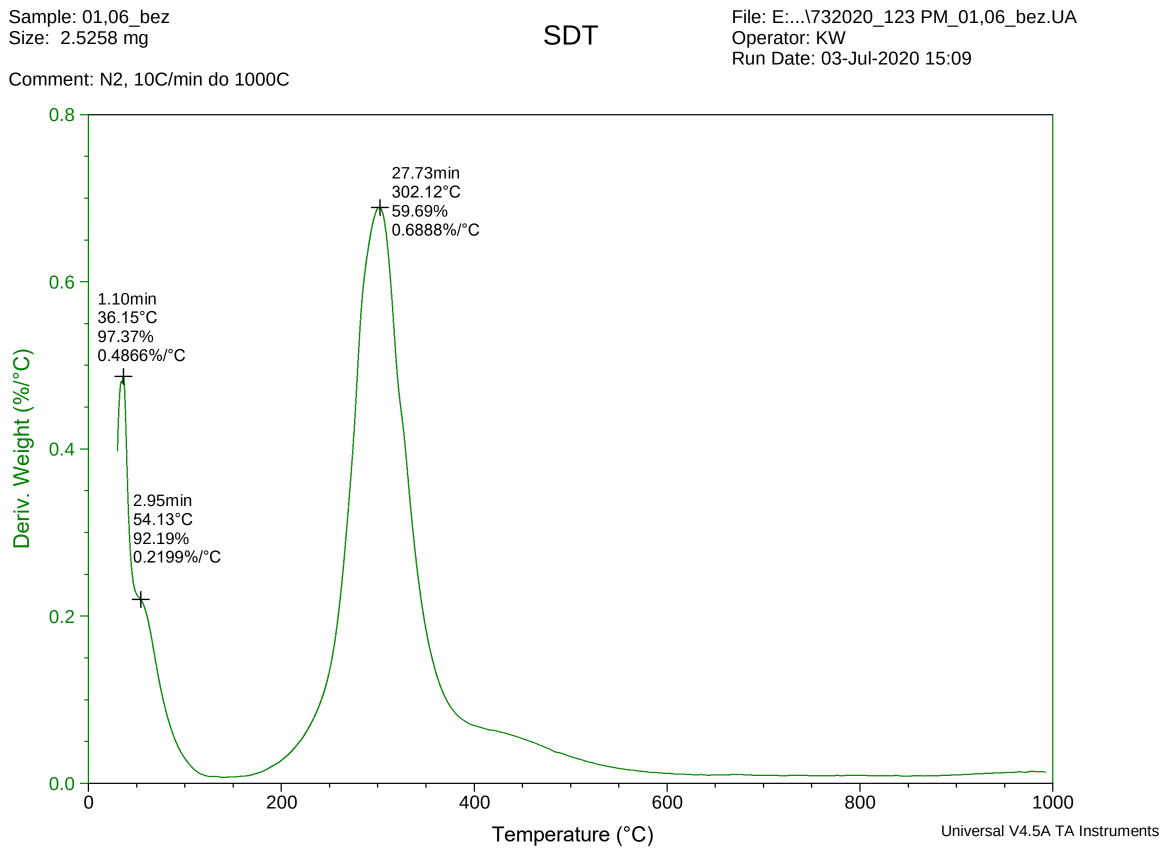

Figure S16. DTA of 1.0 carboxymethyl starch : 1.0 chitosan.

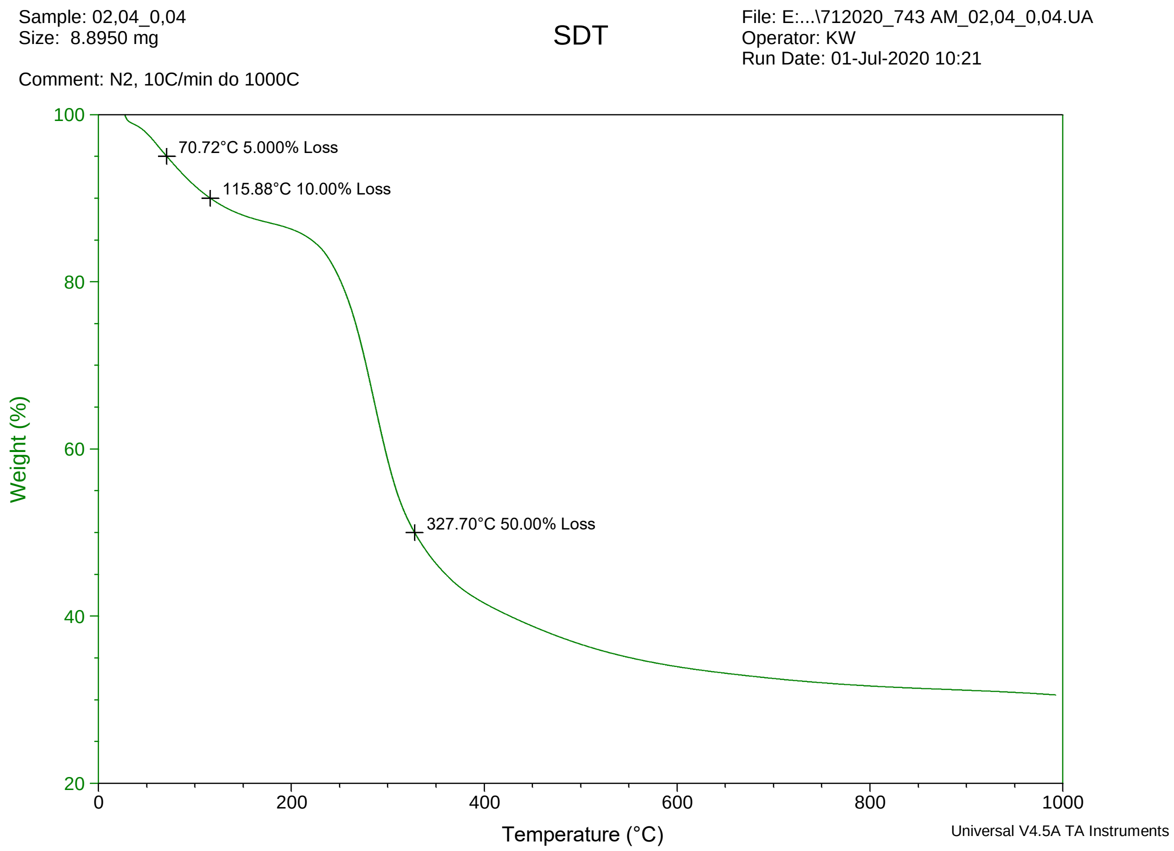

Figure S17. TGA of 1.0 carboxymethyl starch : 1.0 chitosan : 0.04 vanillin.

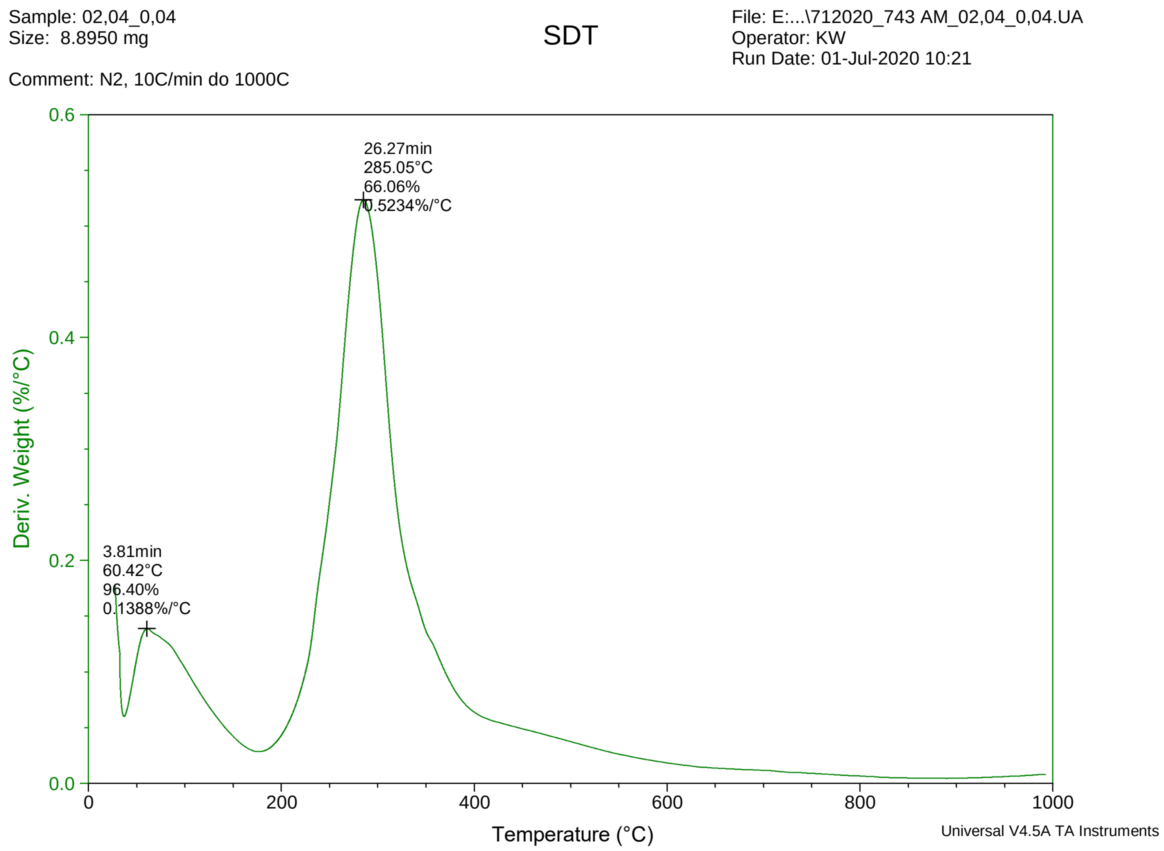

Figure S18. DTA of 1.0 carboxymethyl starch : 1.0 chitosan : 0.04 vanillin.

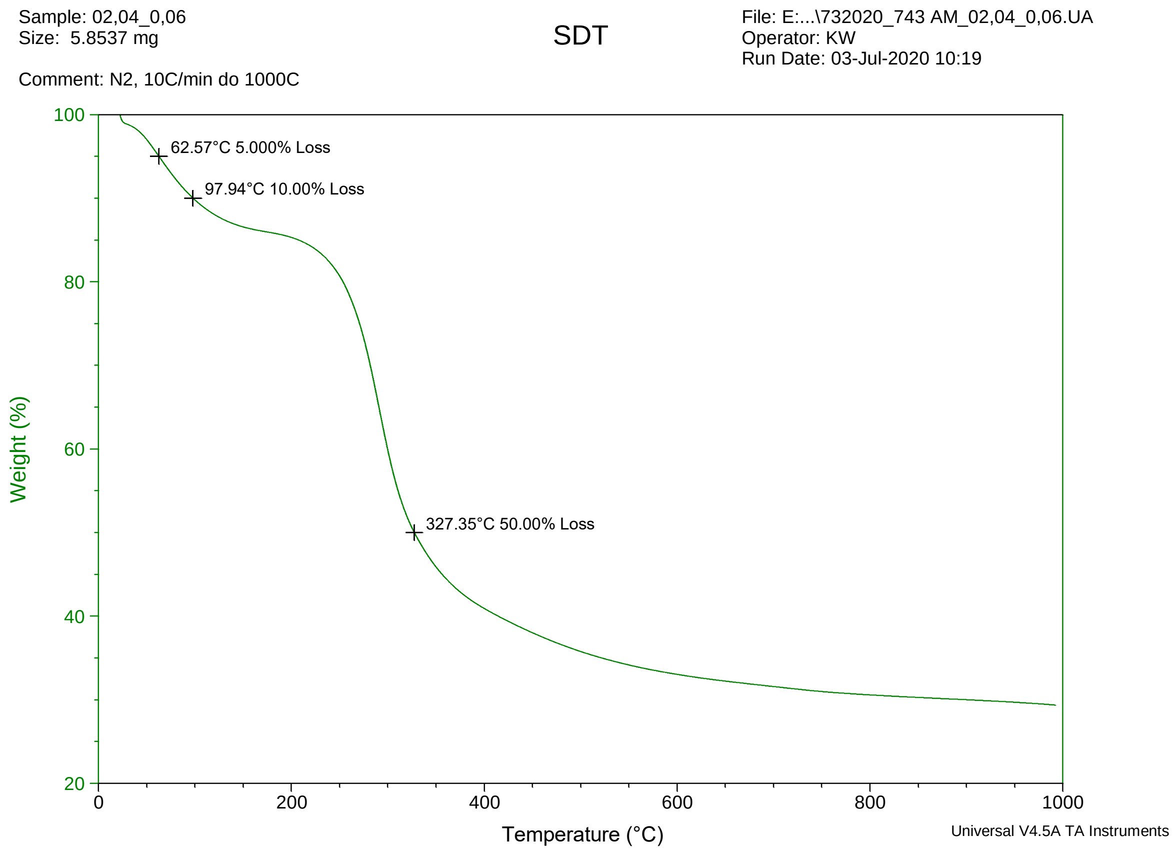

Figure S19. TGA of 1.0 carboxymethyl starch : 1.0 chitosan : 0.06 vanillin.

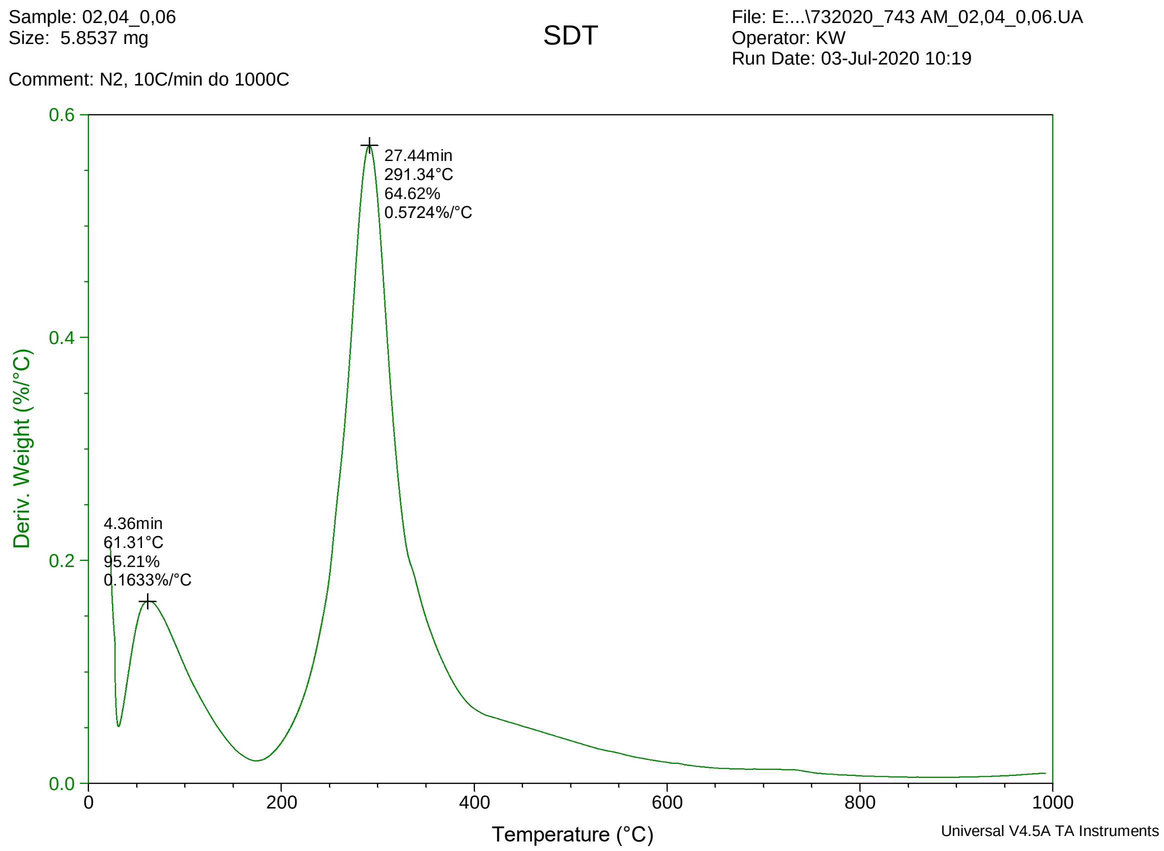

Figure S20. DTA of 1.0 carboxymethyl starch : 1.0 chitosan : 0.06 vanillin.

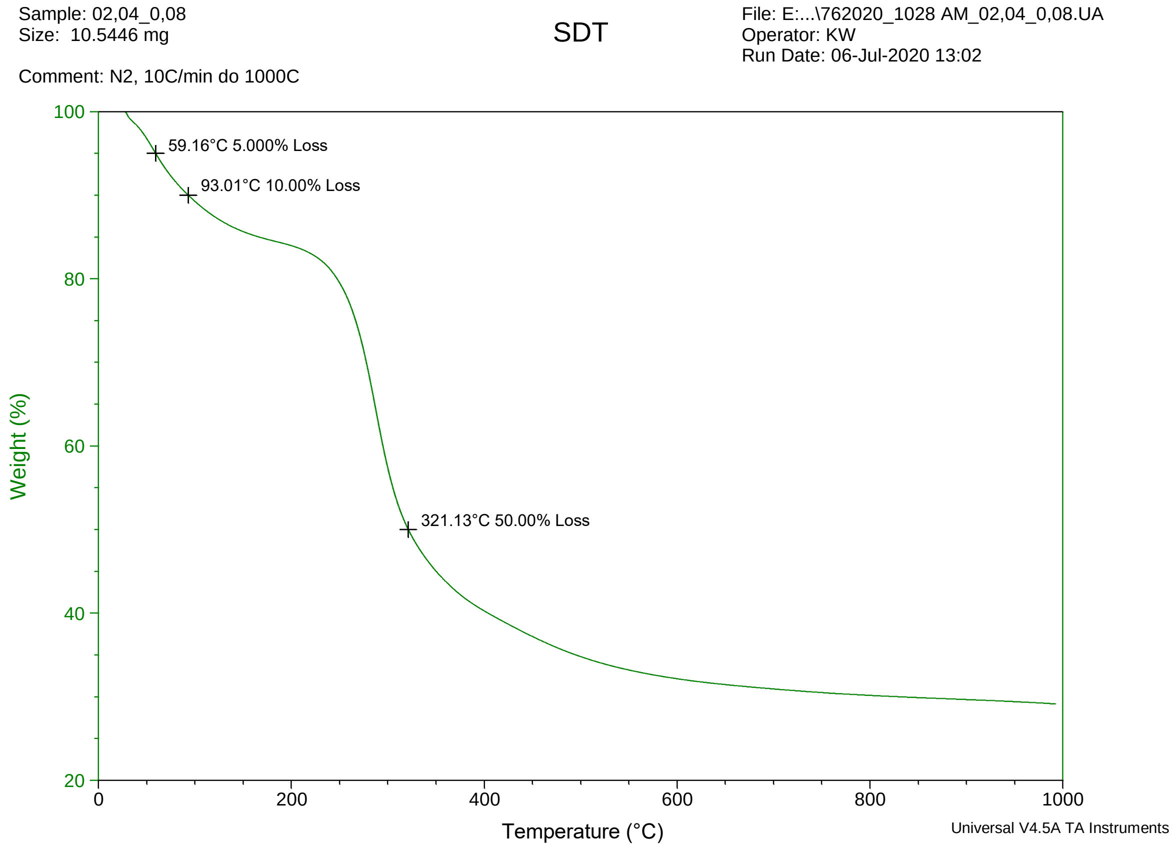

Figure S21. TGA of 1.0 carboxymethyl starch : 1.0 chitosan : 0.08 vanillin.

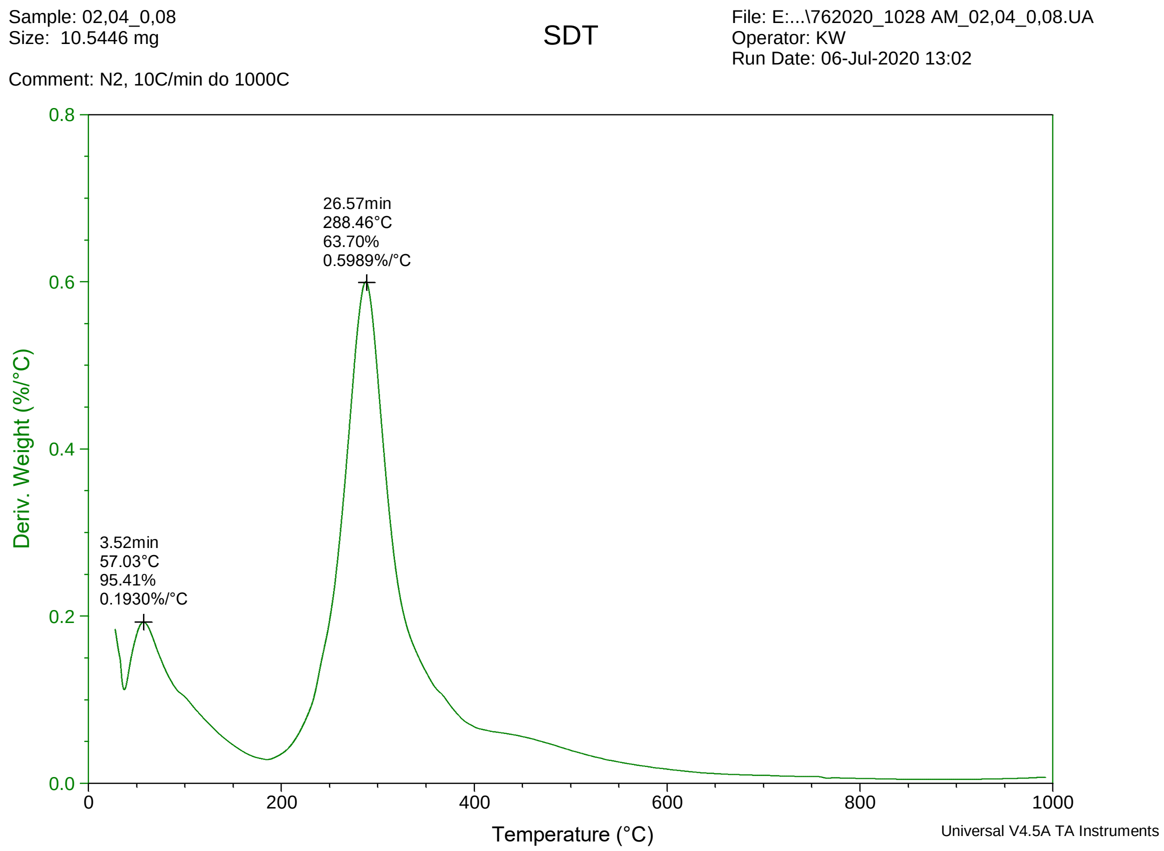

Figure S22. DTA of 1.0 carboxymethyl starch : 1.0 chitosan : 0.08 vanillin.

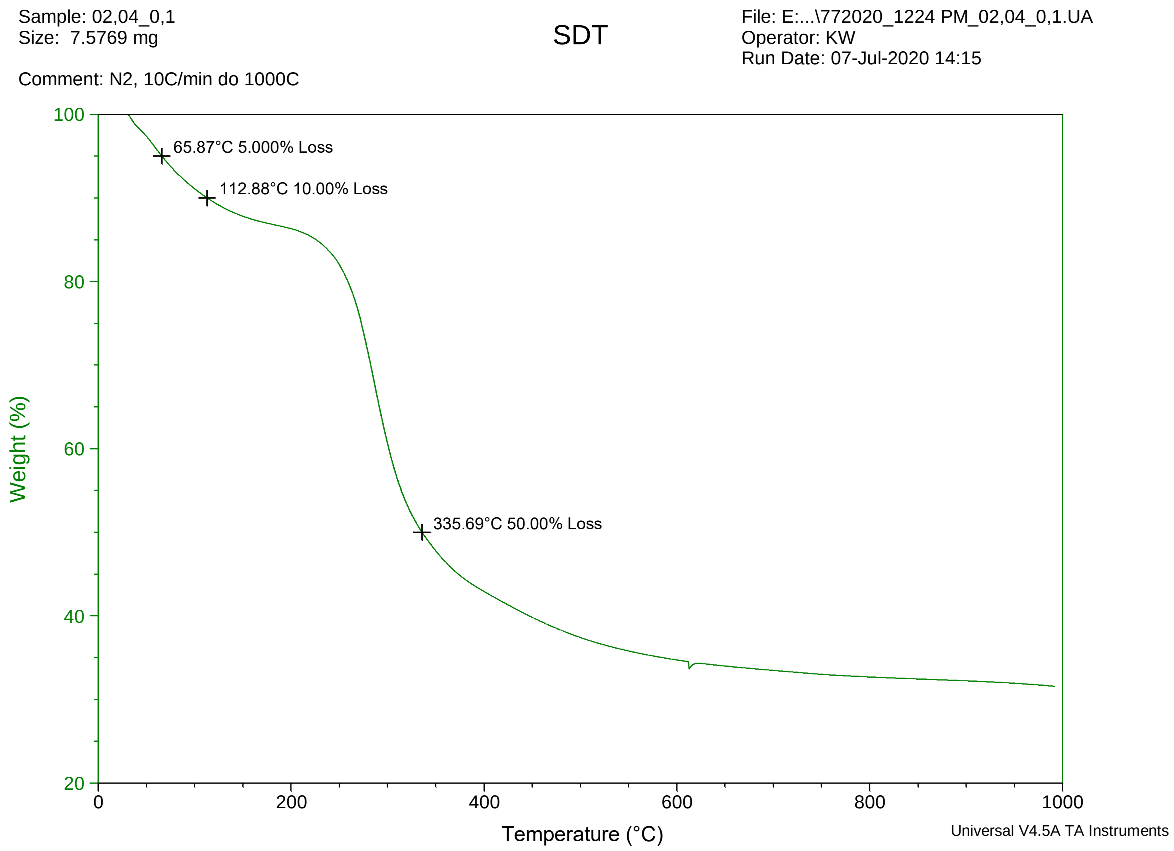

Figure S23. TGA of 1.0 carboxymethyl starch : 1.0 chitosan : 0.10 vanillin.

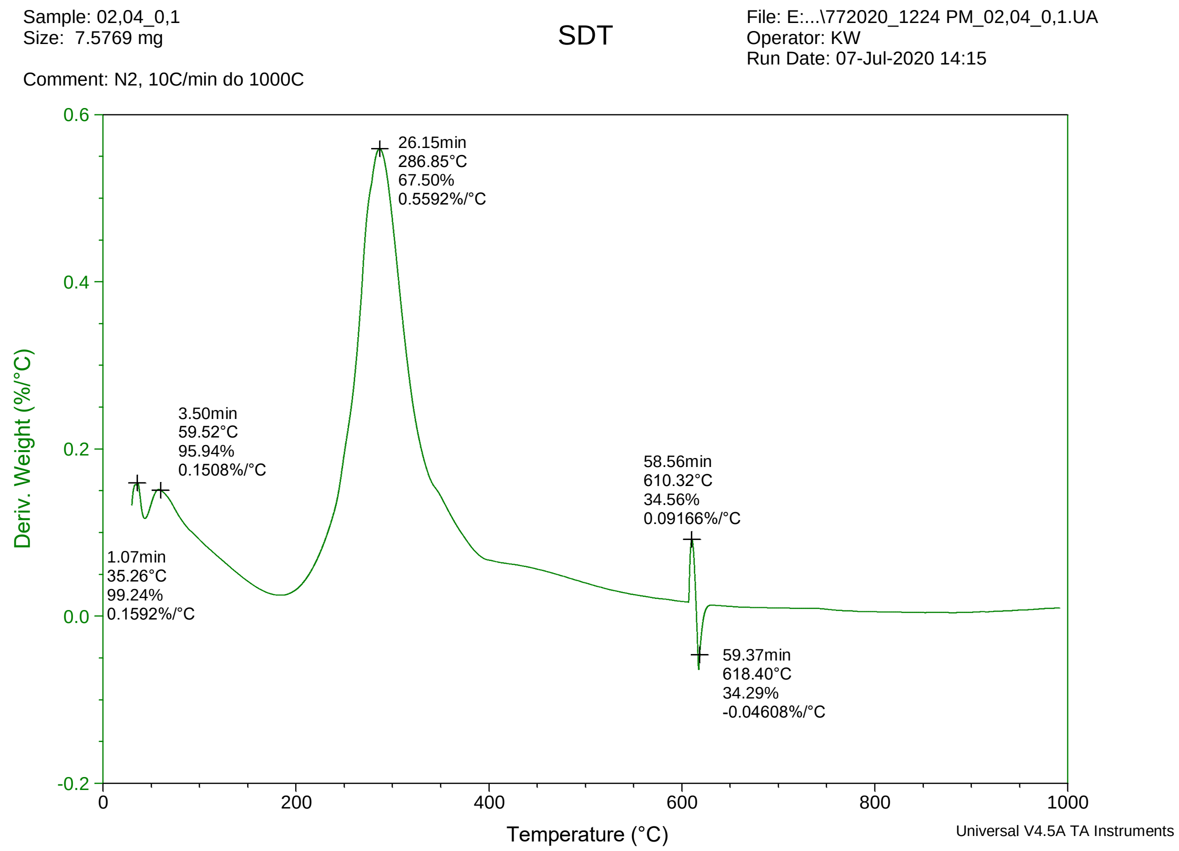

Figure S24. DTA of 1.0 carboxymethyl starch : 1.0 chitosan : 0.10 vanillin.

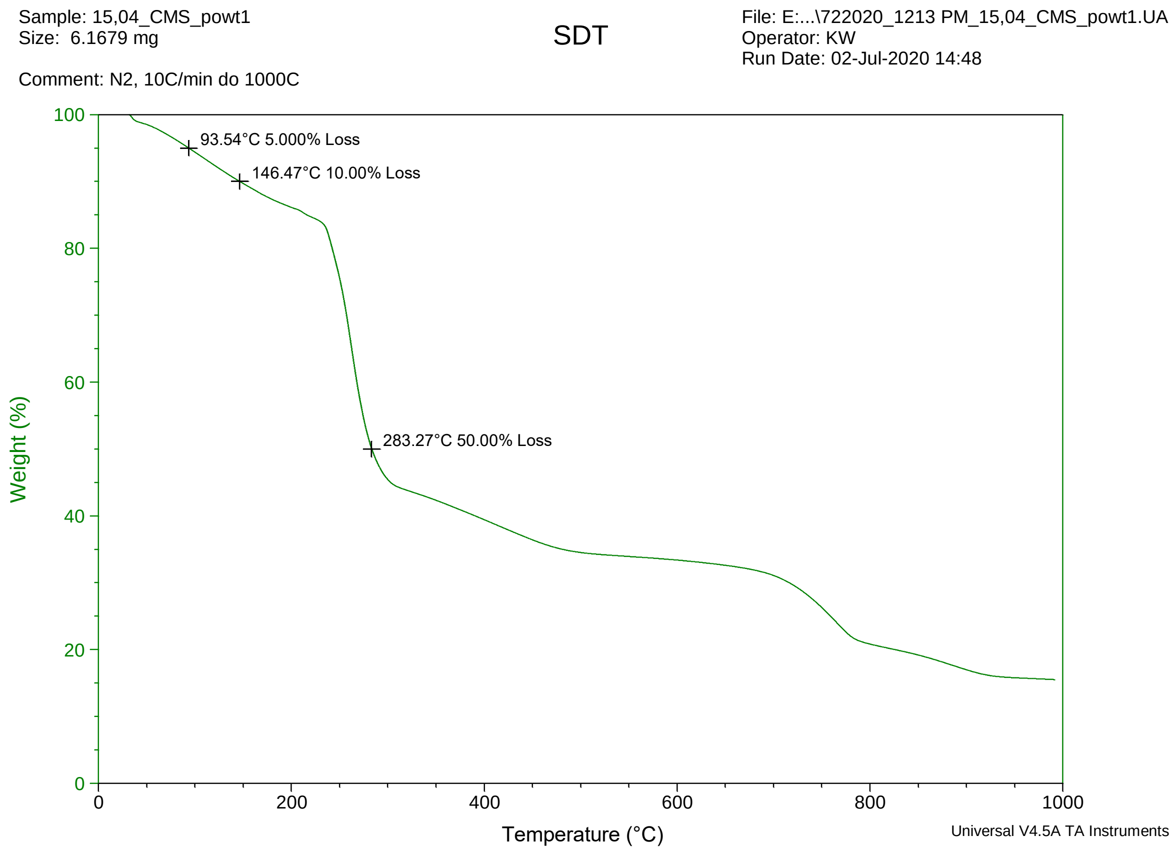

Figure S25. TGA of carboxymethyl starch (CMS).

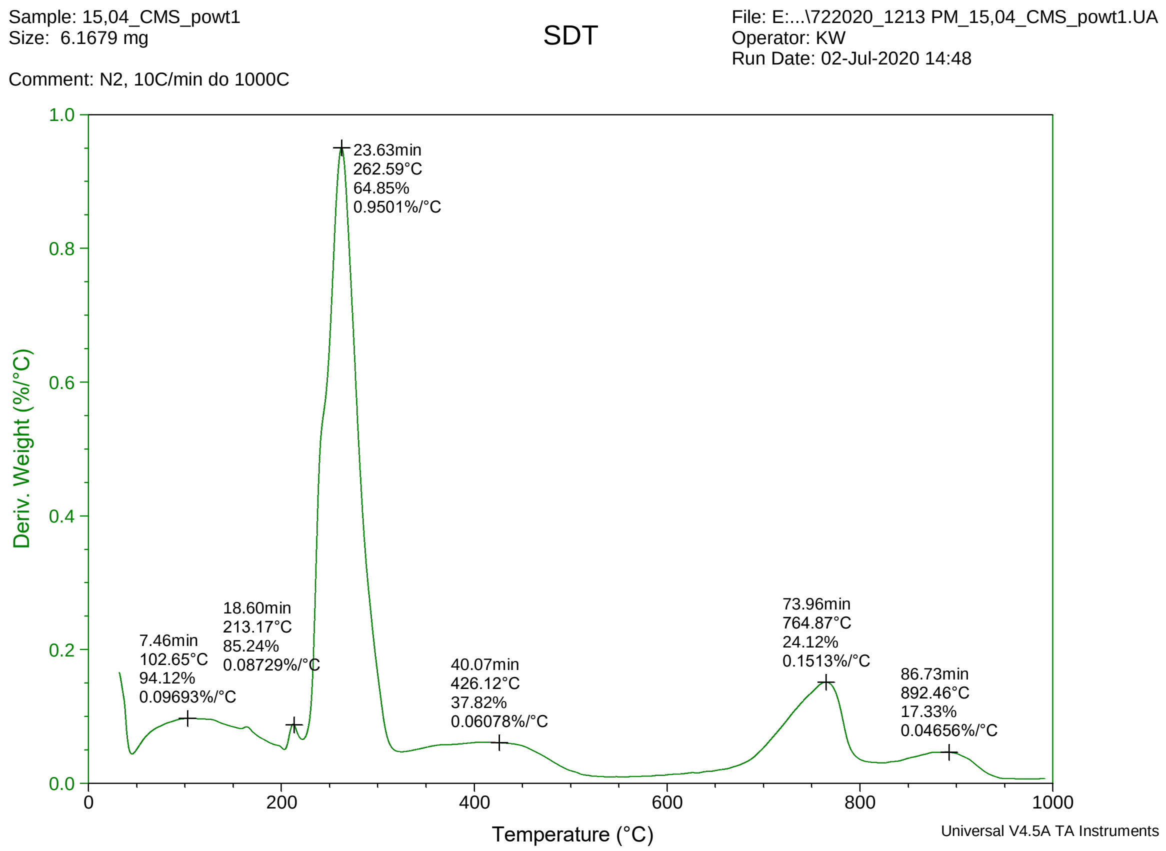

Figure S26. DTA of carboxymethyl starch (CMS).

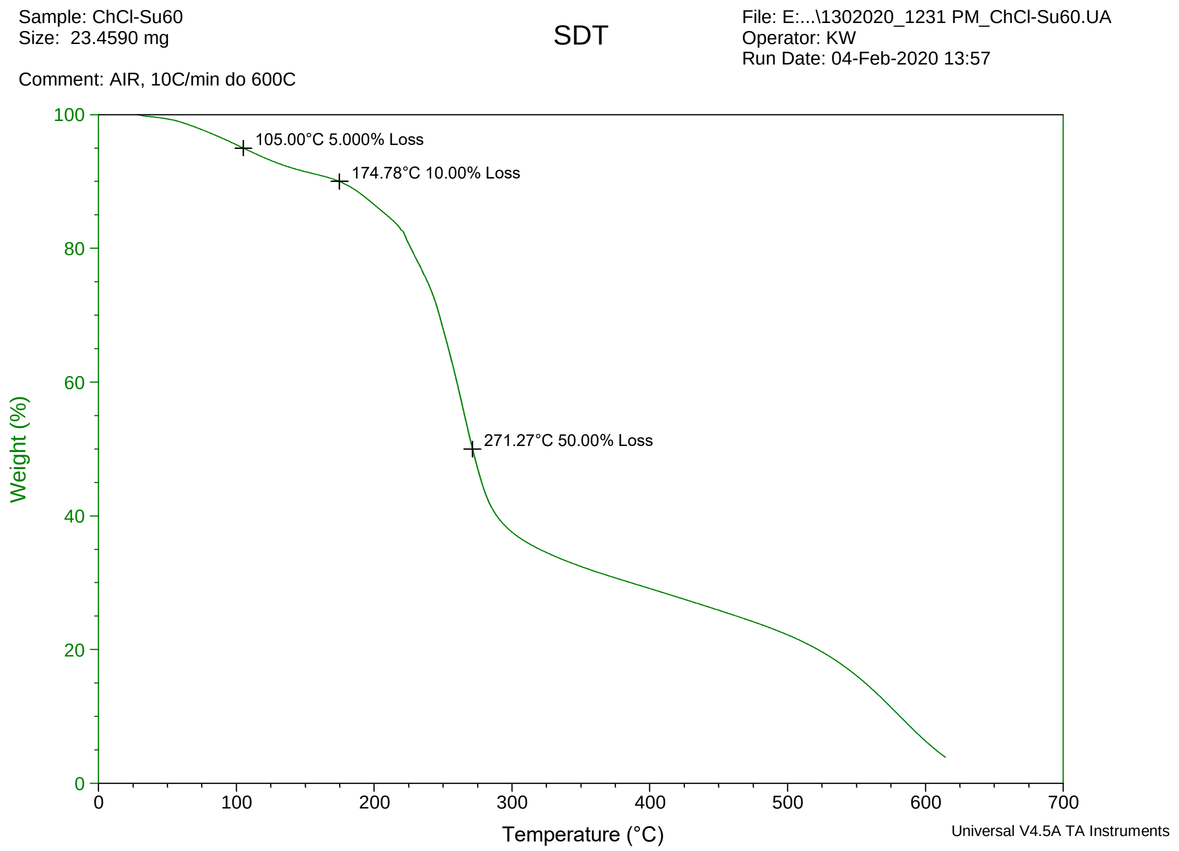

Figure S27. TGA of chitosan (Ch).

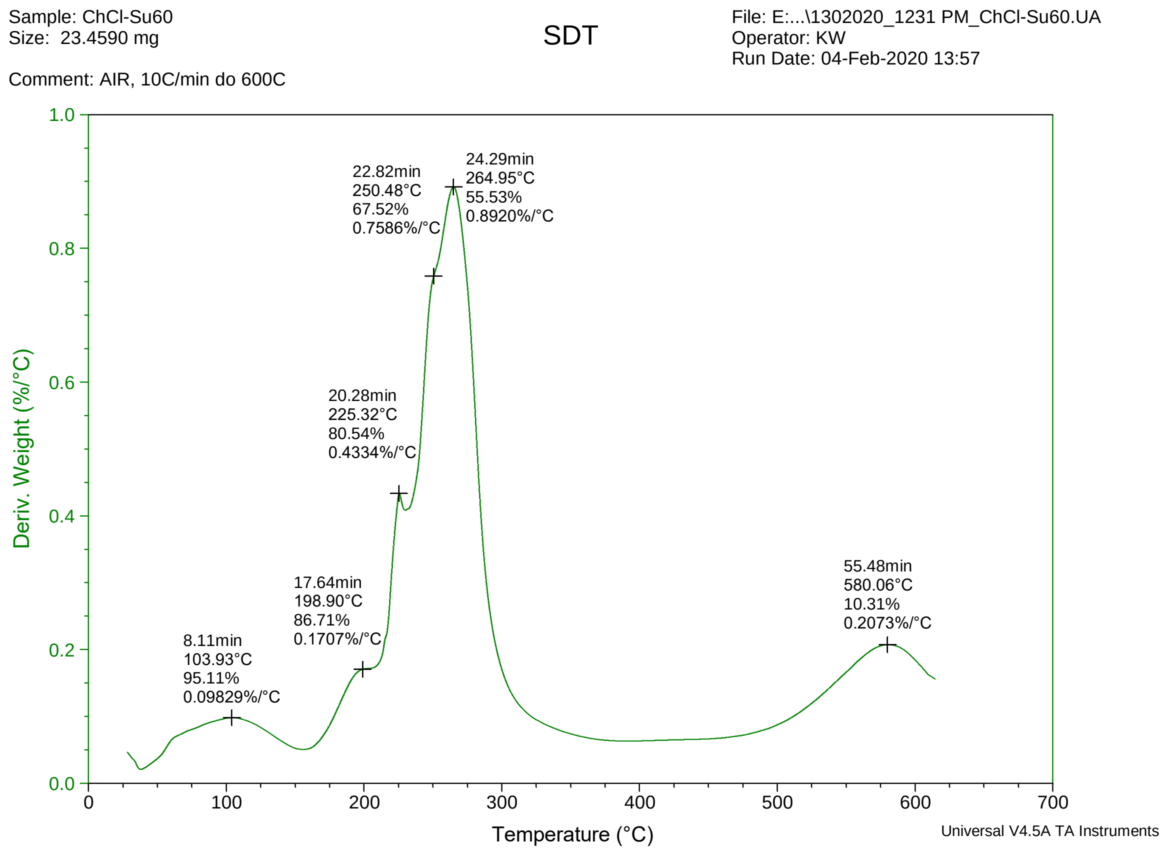

Figure S28. DTA of chitosan (Ch).

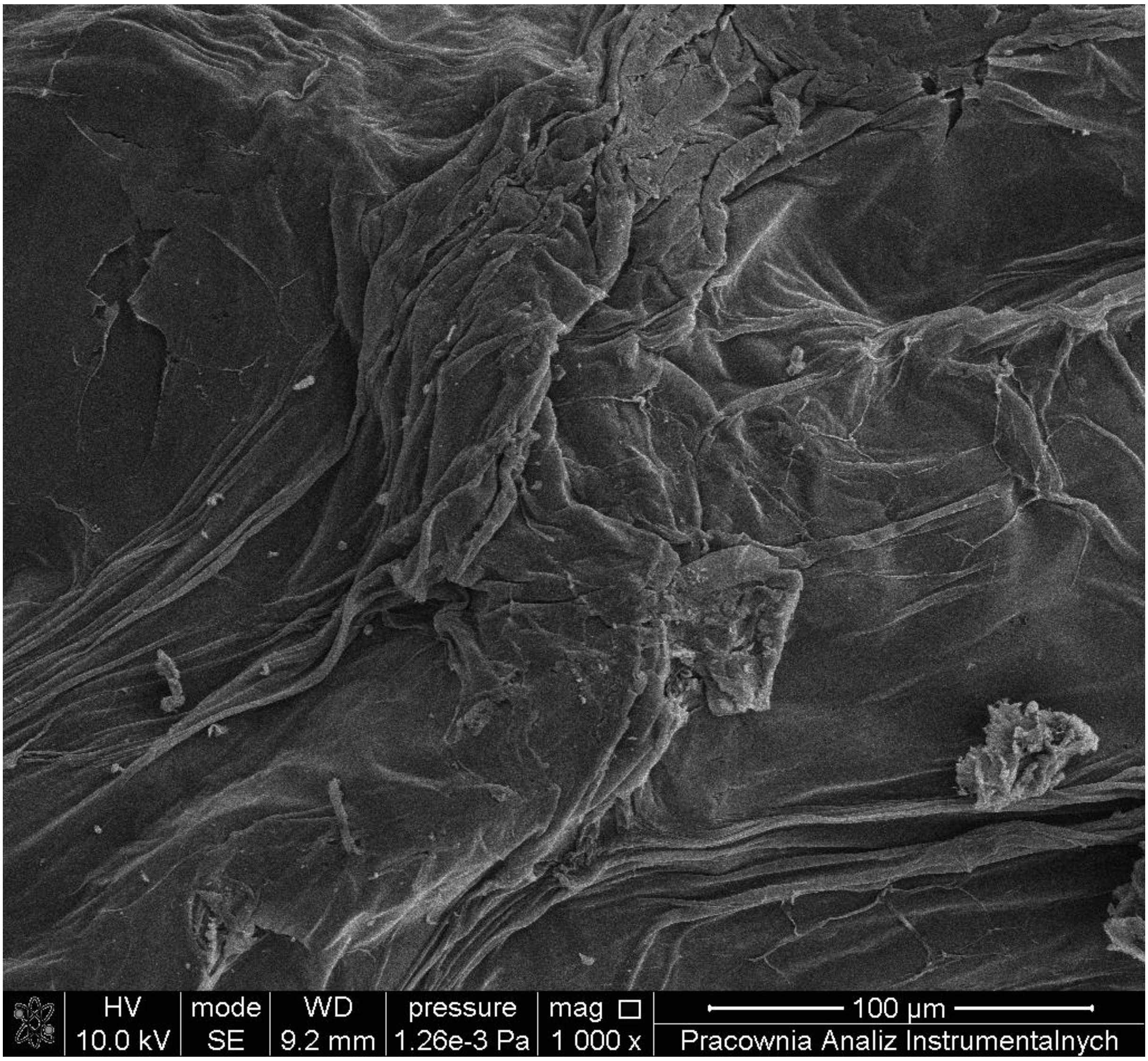

Figure S29. The SEM micrographs with 1000x magnification of CMS-g-Ch without vanillin.

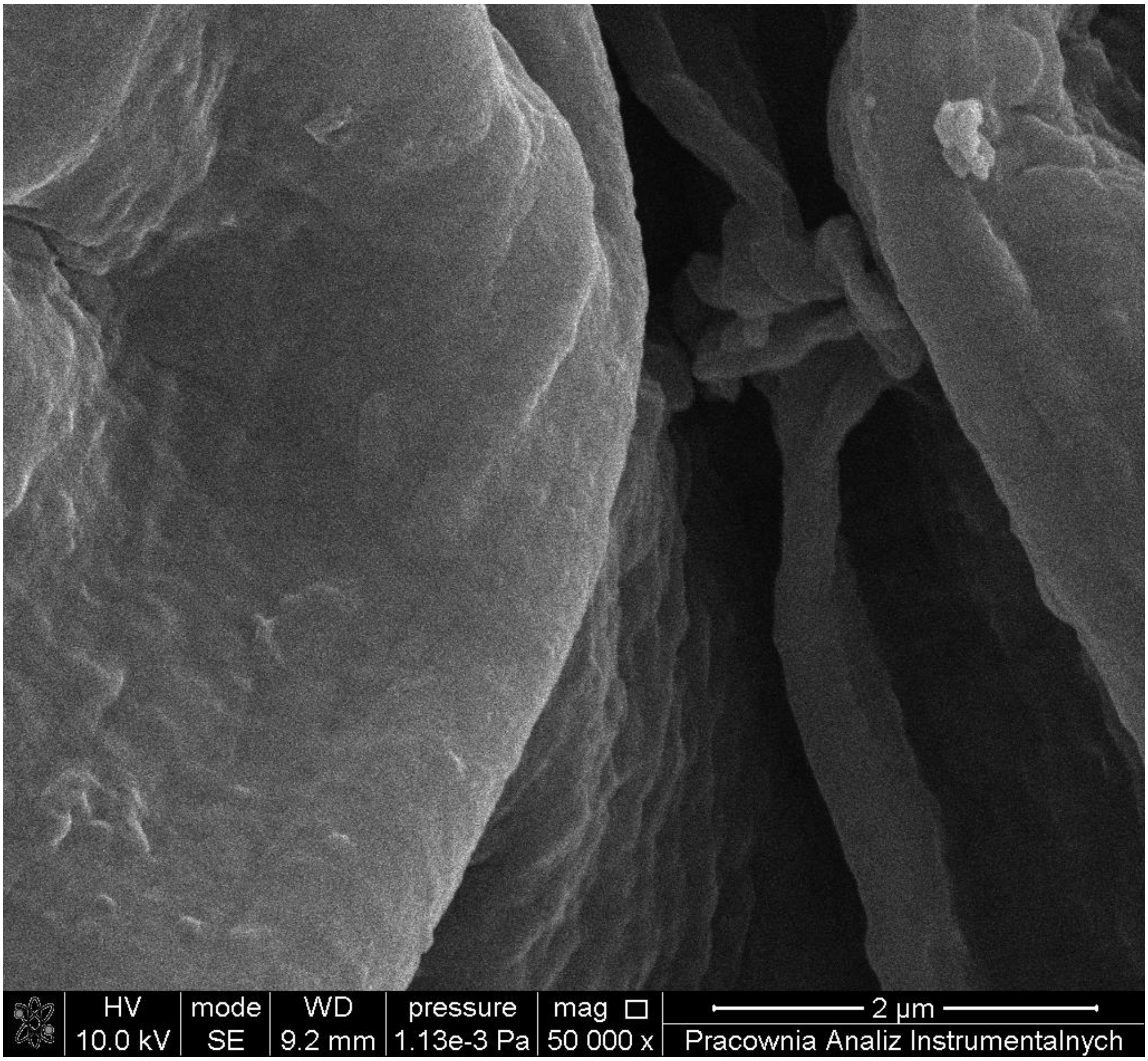

Figure S30. The SEM micrographs with 50000x magnification of CMS-g-Ch without vanillin.

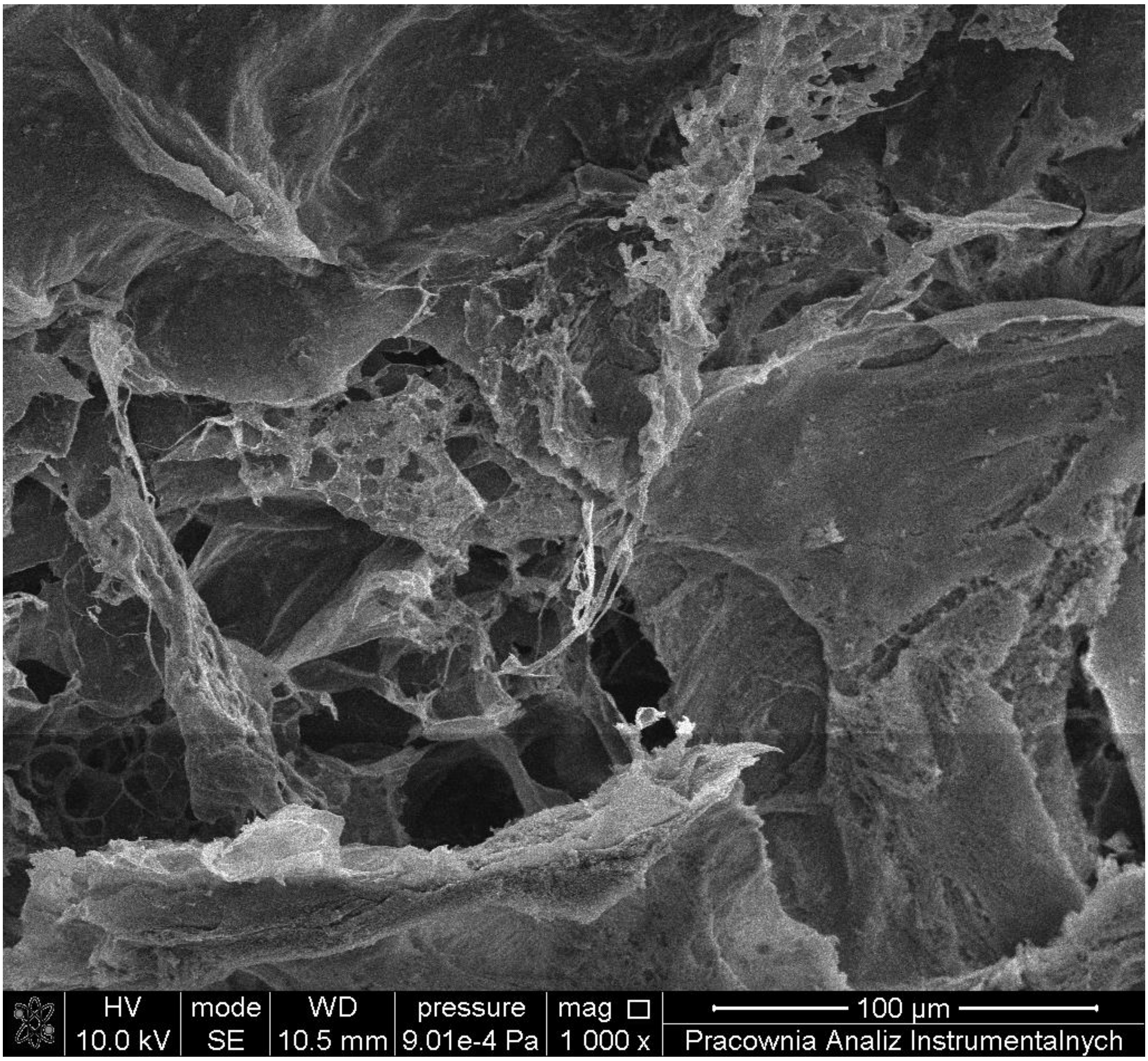

Figure S31. The SEM micrographs with 1000x magnification of CMS-g-Ch with 0,04 vanillin.

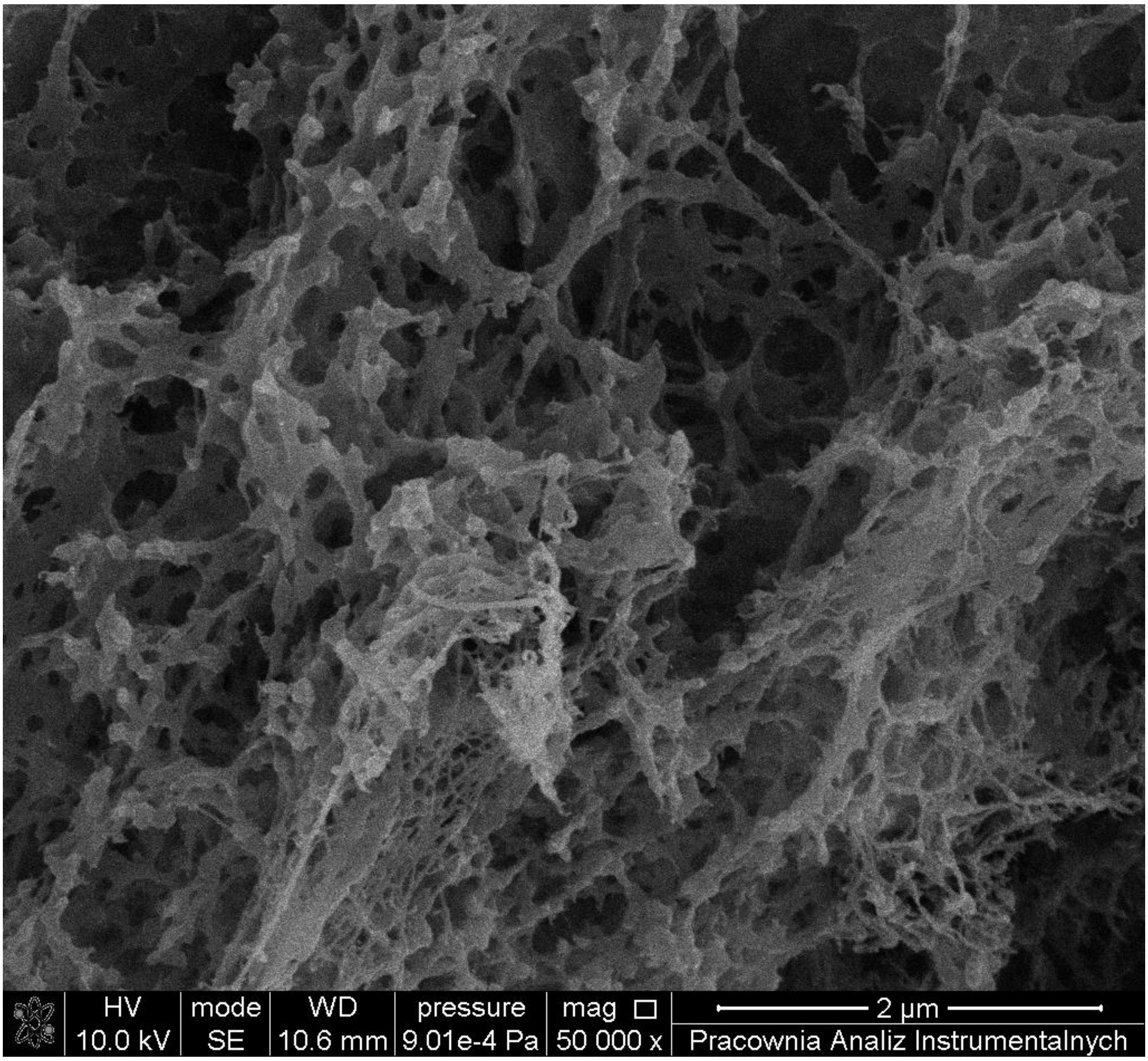

Figure S32. The SEM micrographs with 50000x magnification of CMS-g-Ch with 0,04 vanillin.

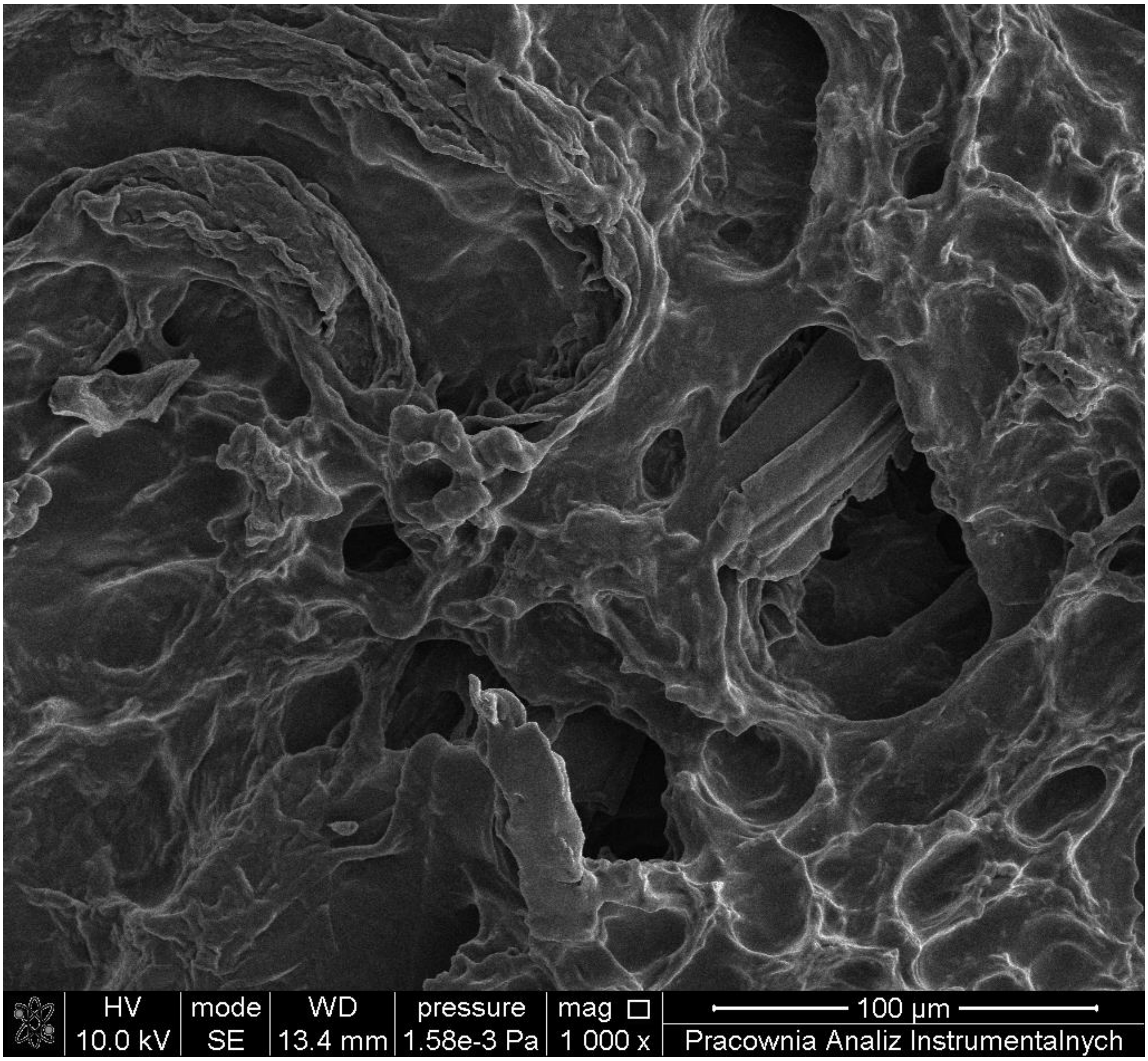

Figure S33. The SEM micrographs with 1000x magnification of CMS-g-Ch with 0,06 vanillin.

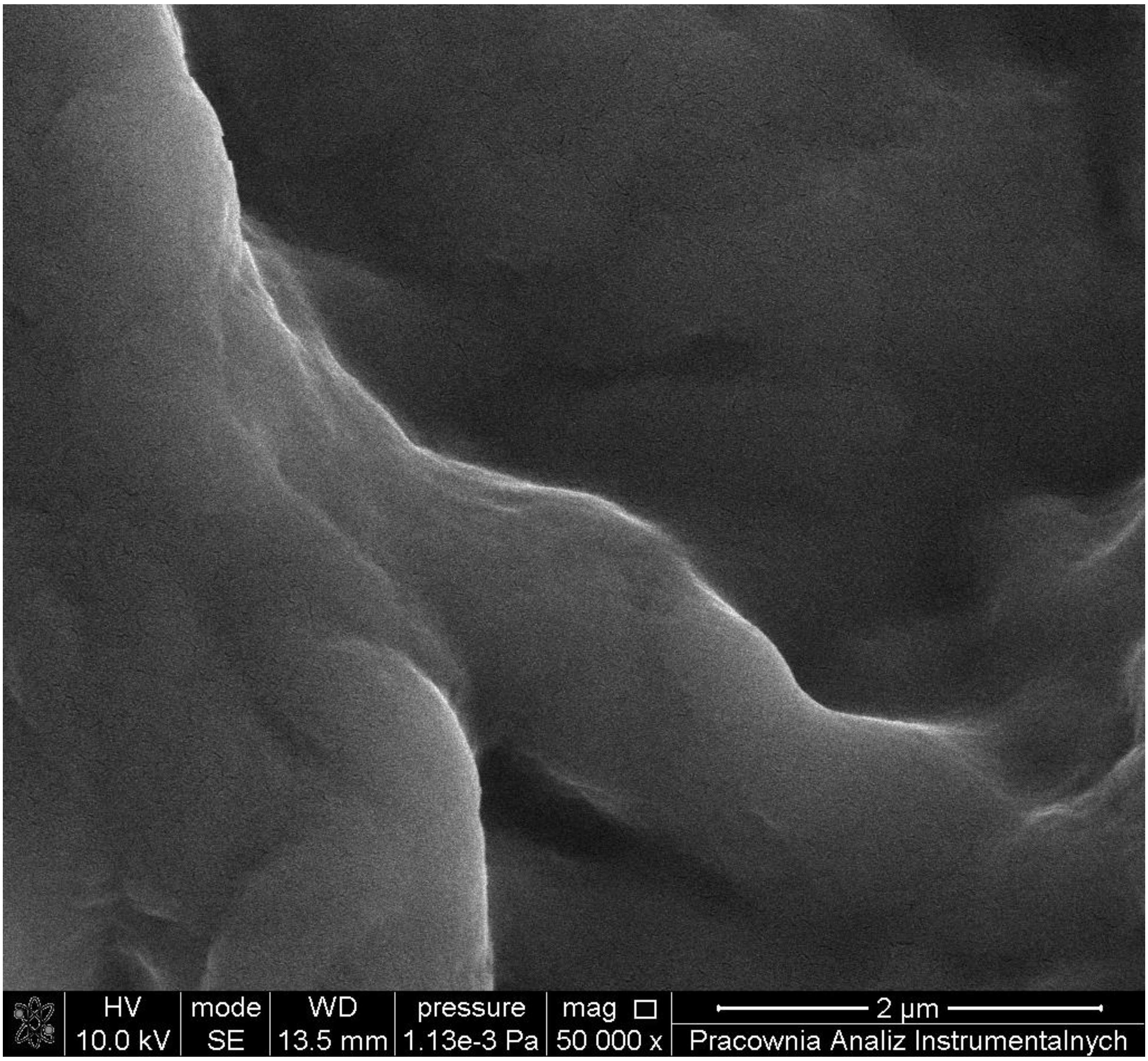

Figure S34. The SEM micrographs with 50000x magnification of CMS-g-Ch with 0,06 vanillin.

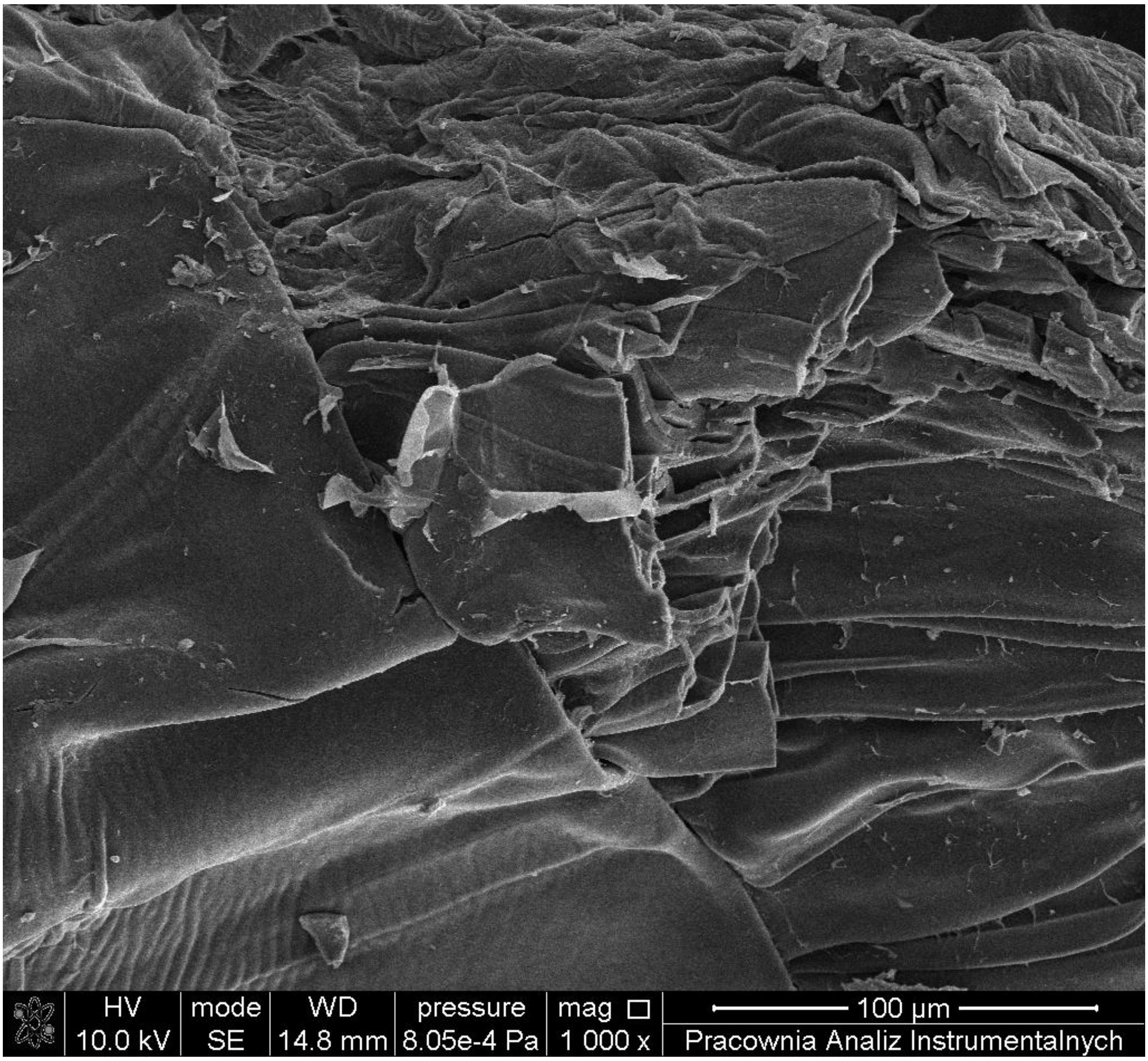

Figure S35. The SEM micrographs with 1000x magnification of CMS-g-Ch with 0,08 vanillin.

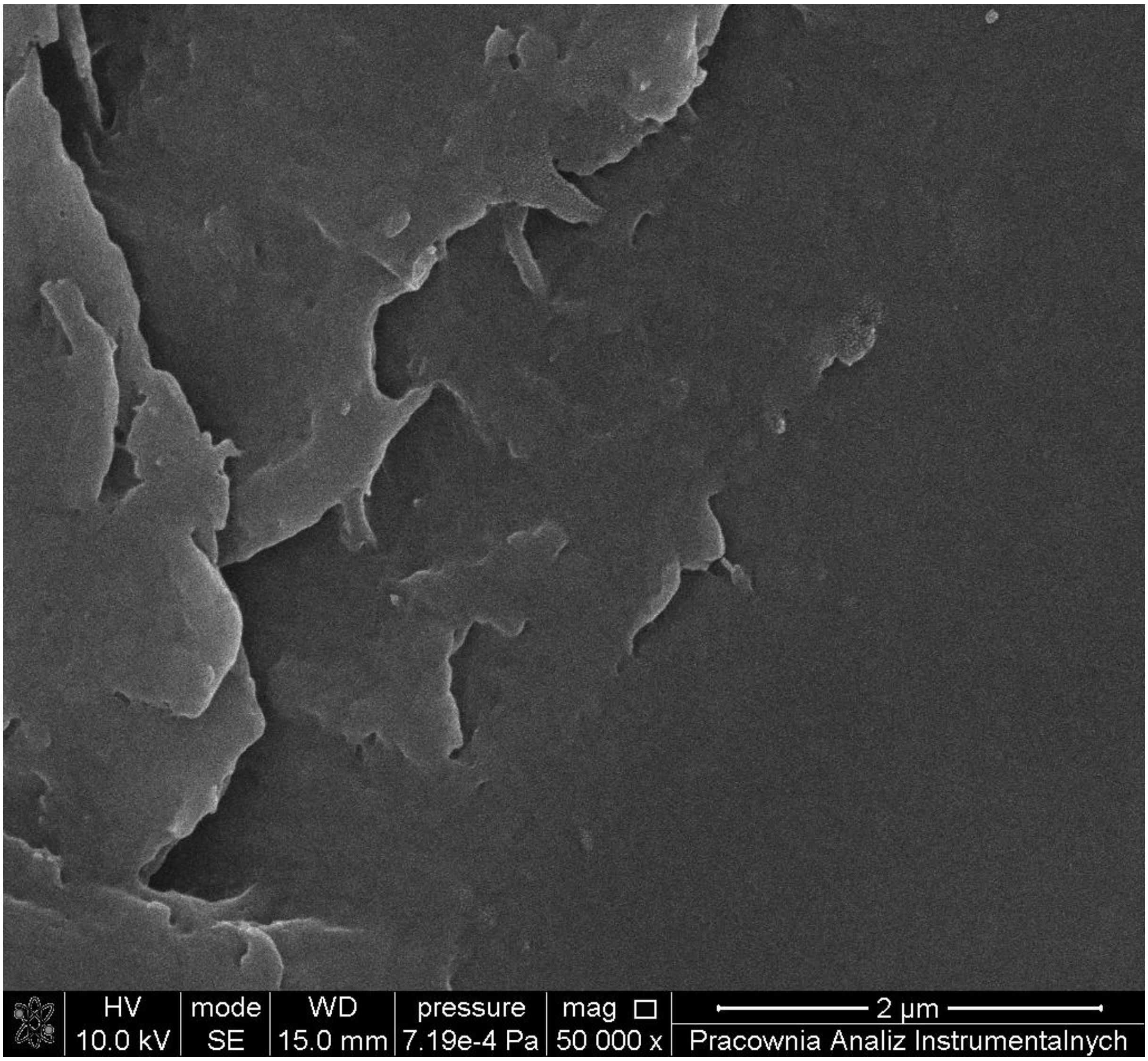

Figure S36. The SEM micrographs with 50000x magnification of CMS-g-Ch with 0,08 vanillin.

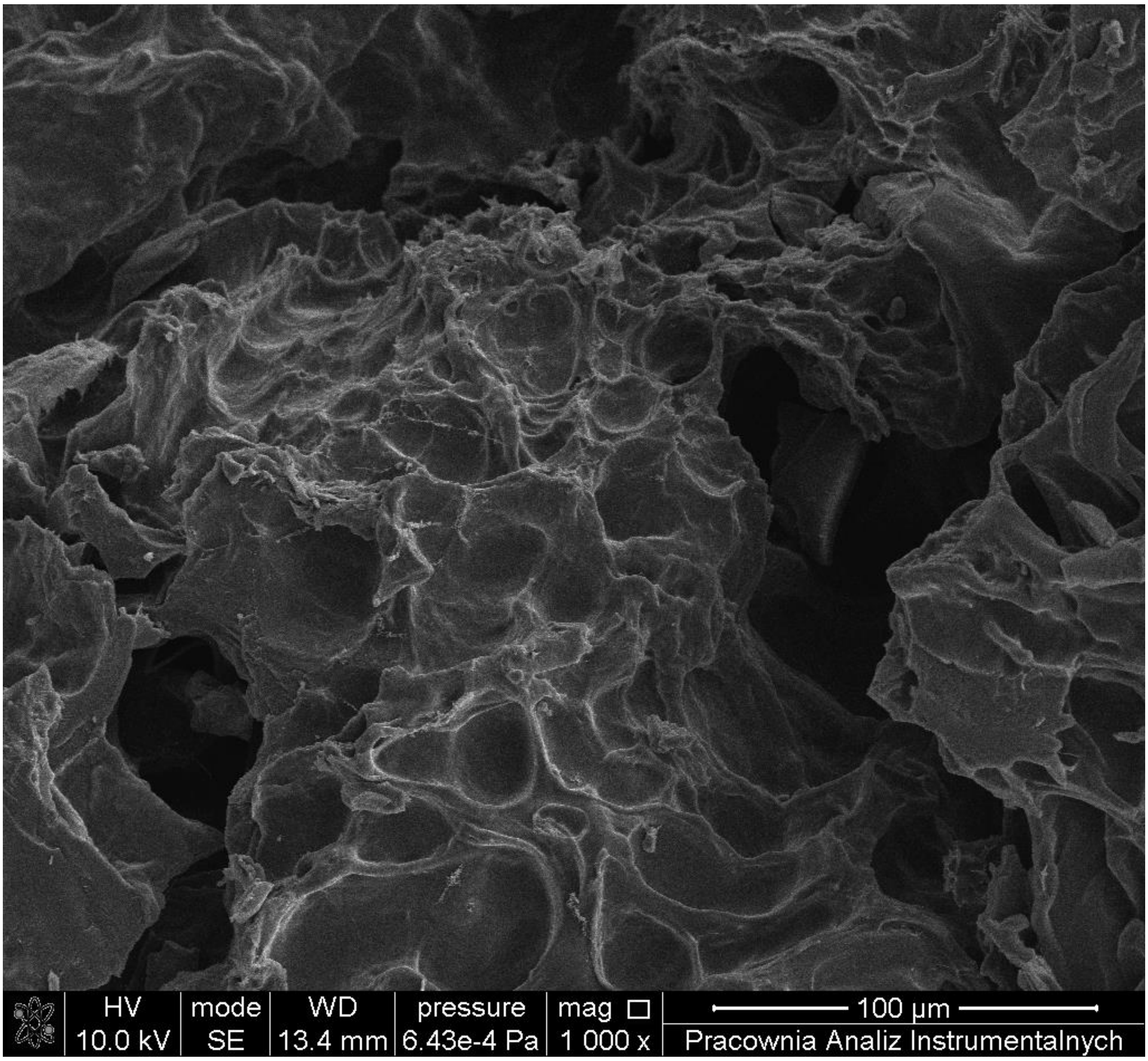

Figure S37. The SEM micrographs with 1000x magnification of CMS-g-Ch with 0,10 vanillin.

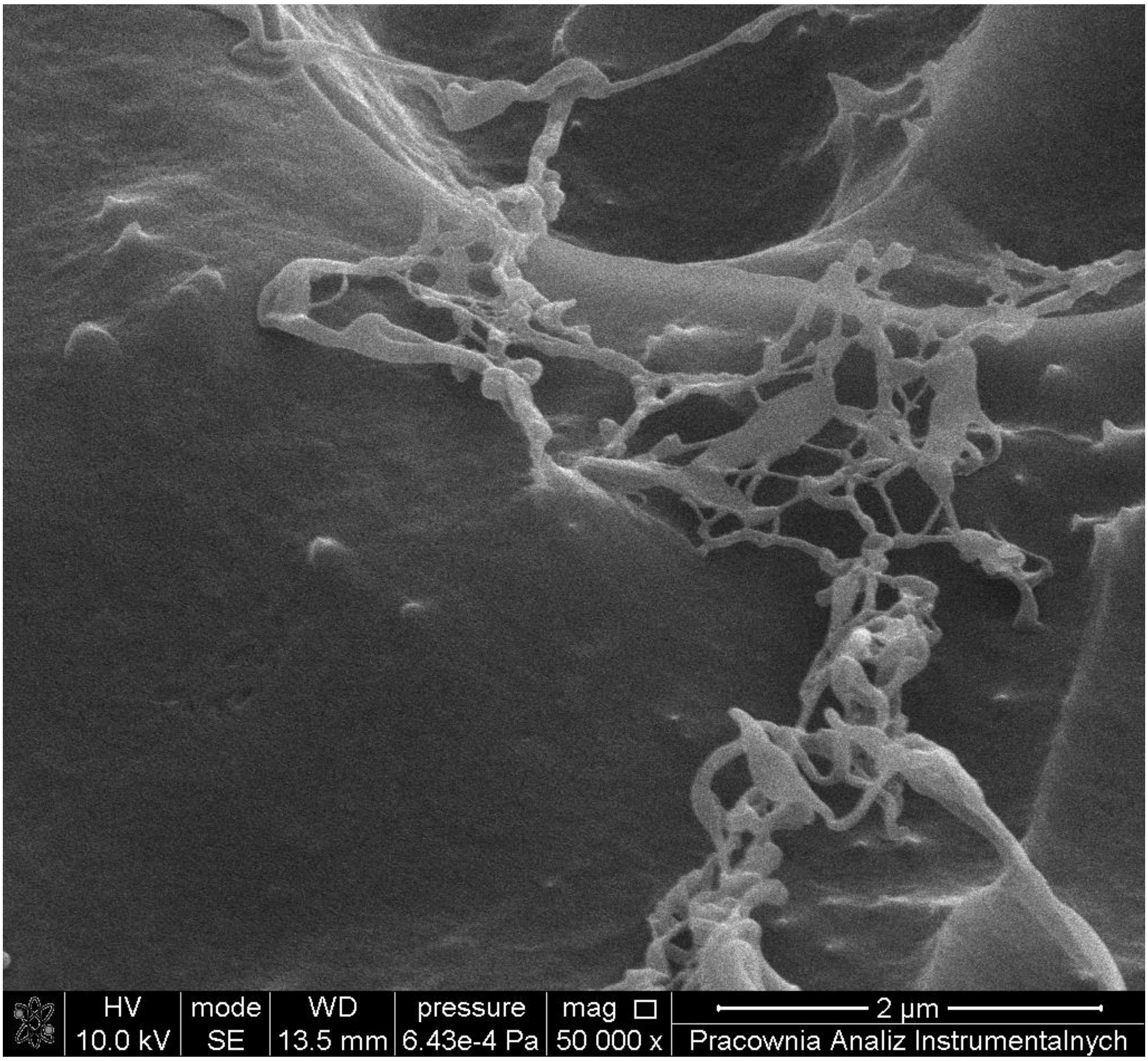

Figure S38. The SEM micrographs with 50000x magnification of CMS-g-Ch with 0,10 vanillin.

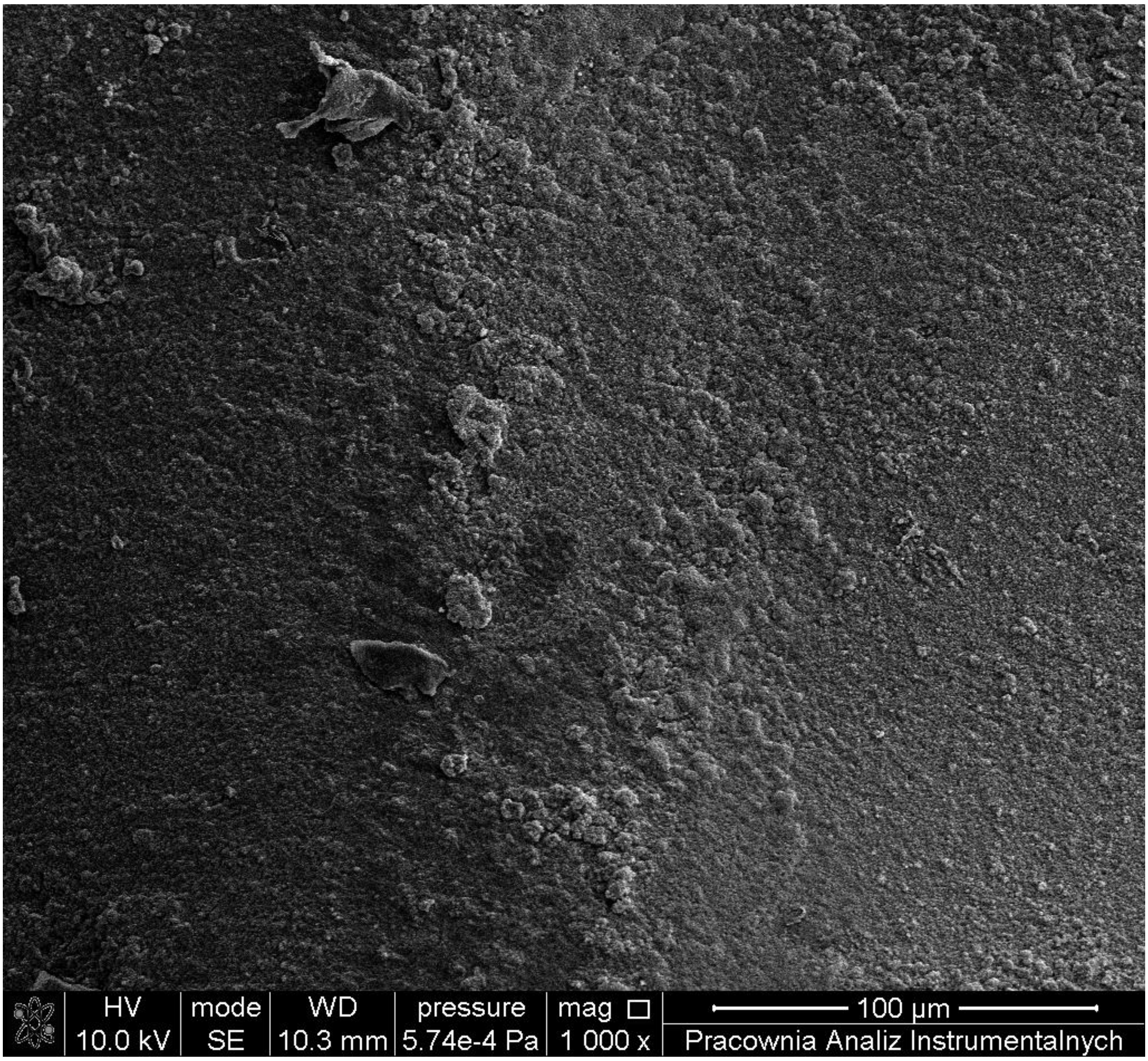

Figure S39. The SEM micrographs with 1000x magnification of CMS.

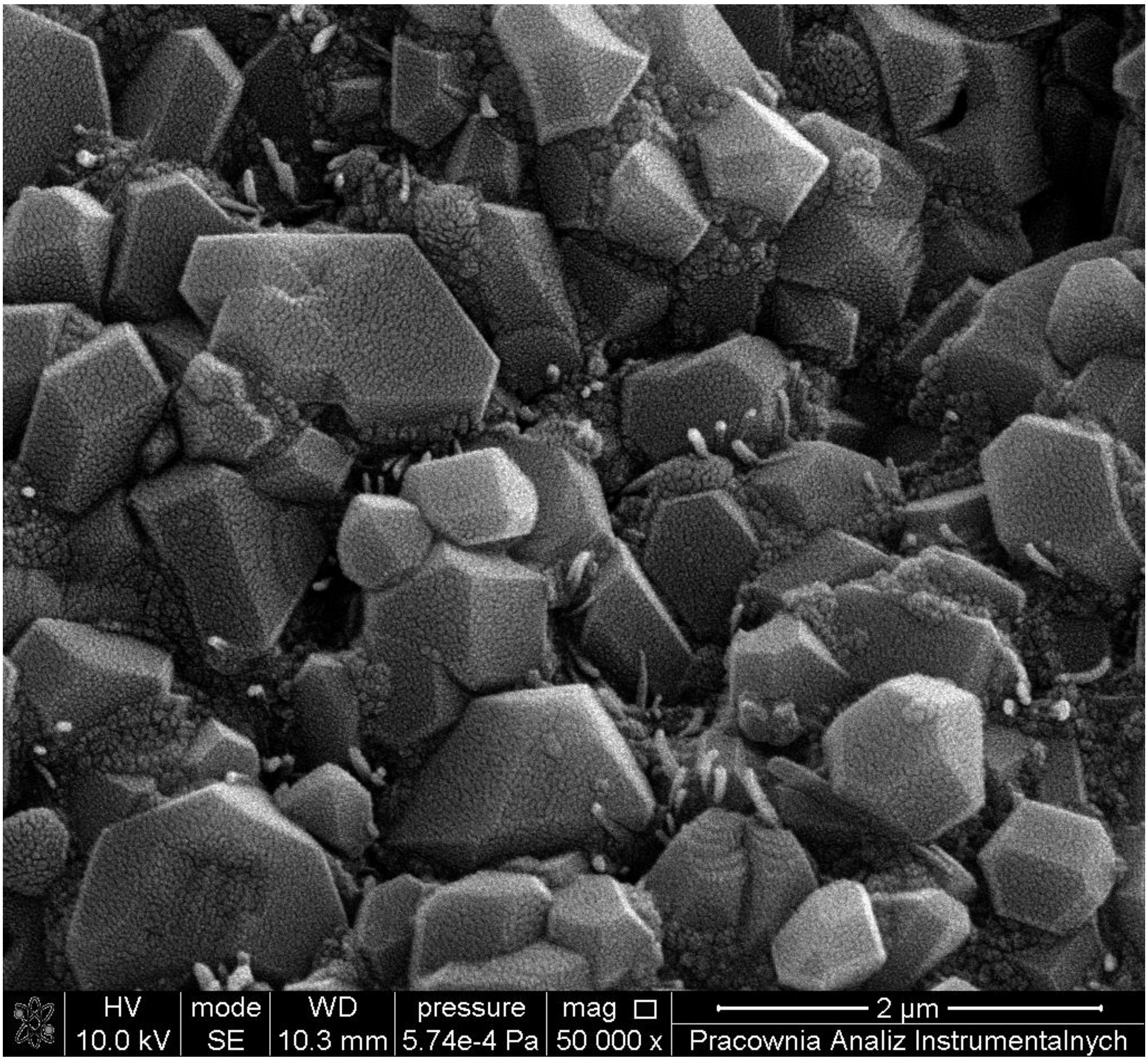

Figure S40. The SEM micrographs with 50000x magnification of CMS.

(a)

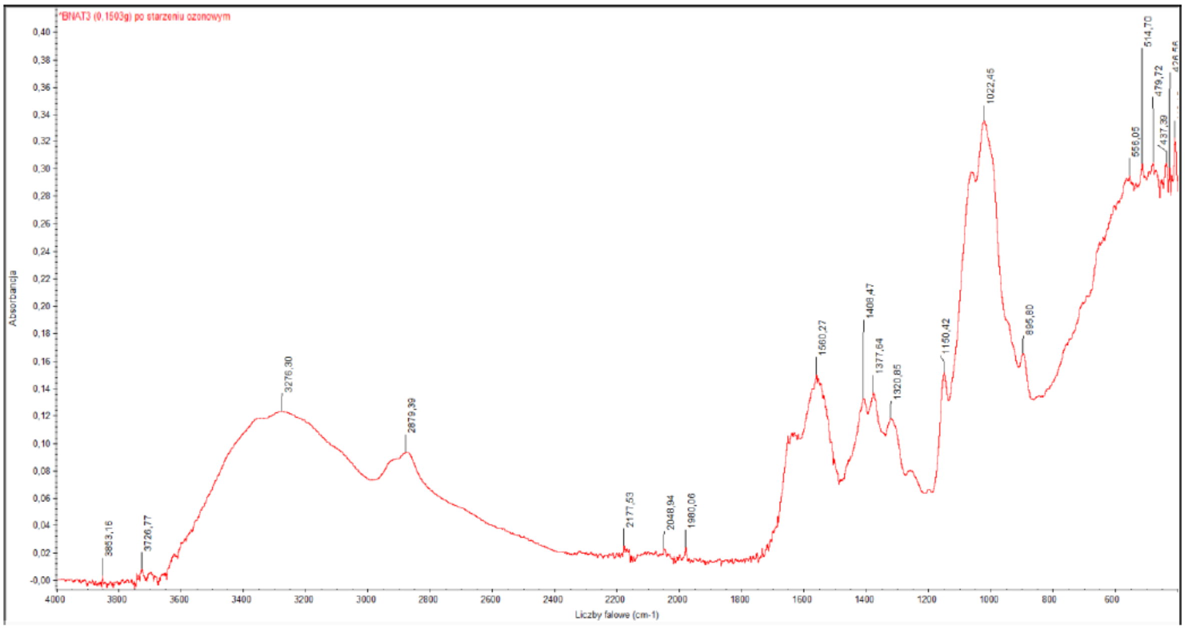

(b)

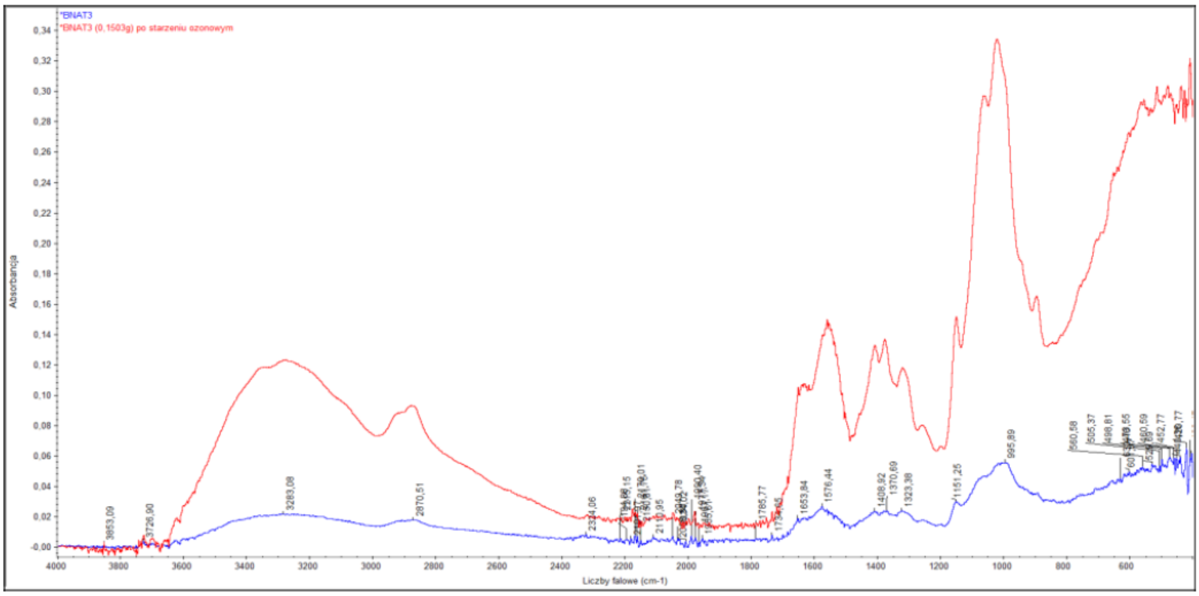

Figure S41. Summary of spectra before (a) and after (b) infrared ozone aging for CMS(1)/Ch(1).

(a)

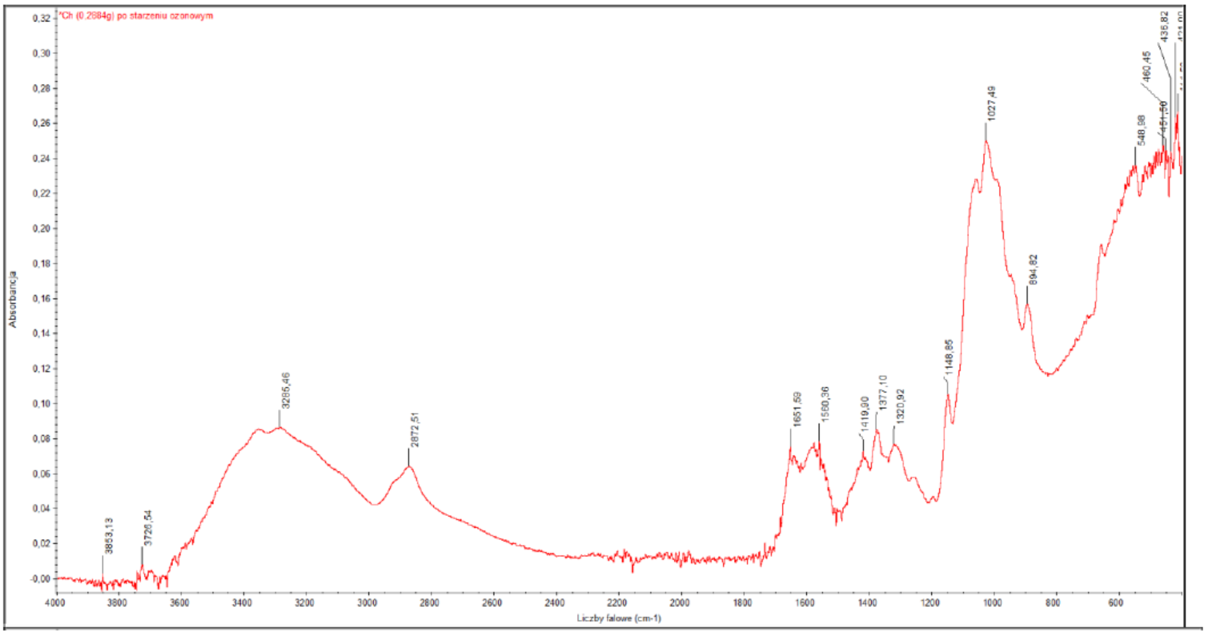

(b)

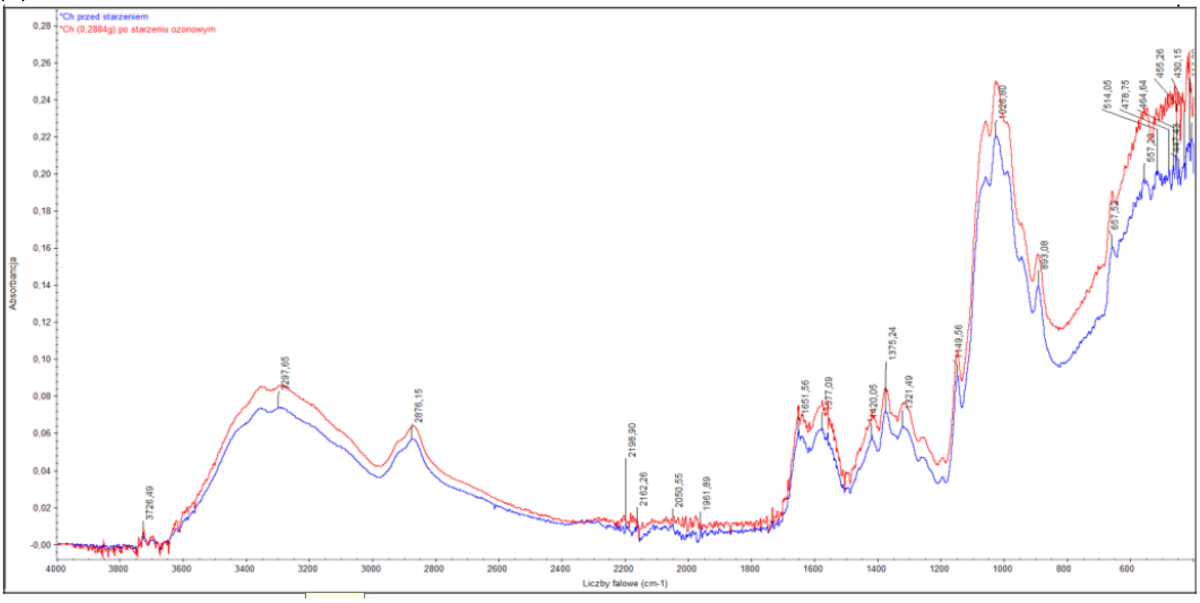

Figure S42. Summary of spectra before (a) and after (b) infrared ozone aging for chitosan (Ch).

(a)

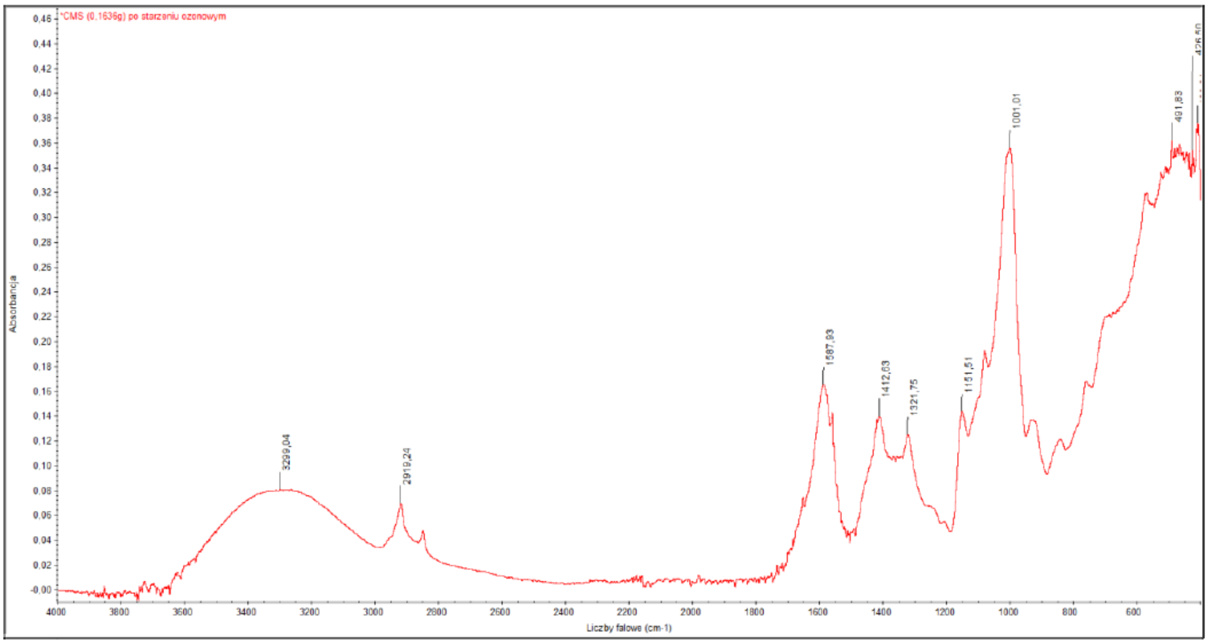

(b)

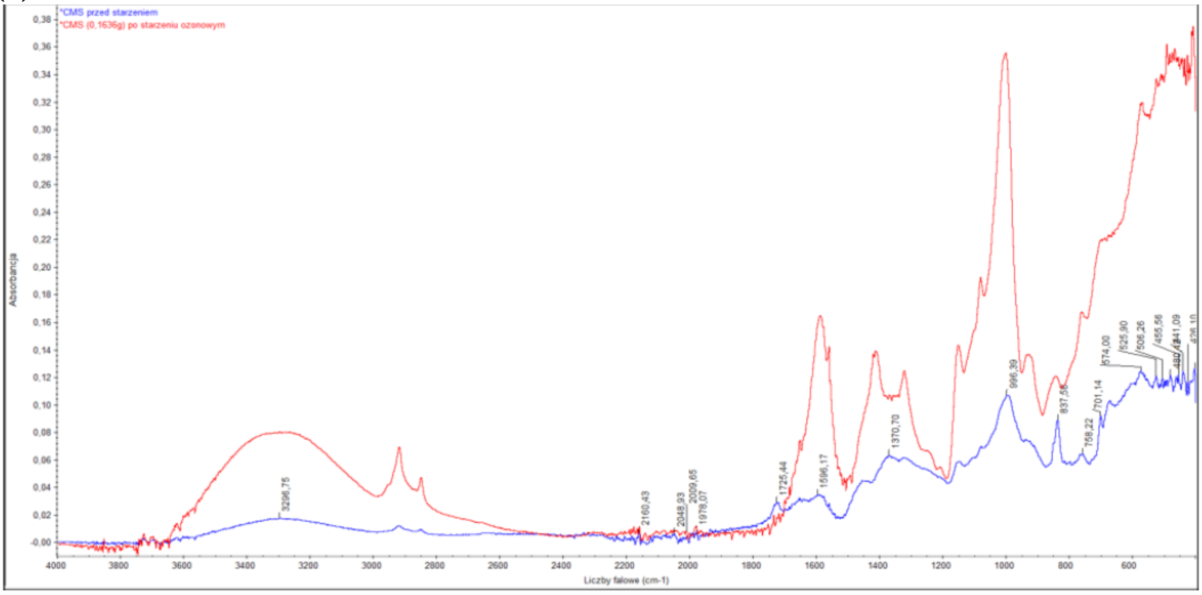

Figure S43. Summary of spectra before (a) and after (b) infrared ozone aging for carboxymethyl starch (CMS).

(a)

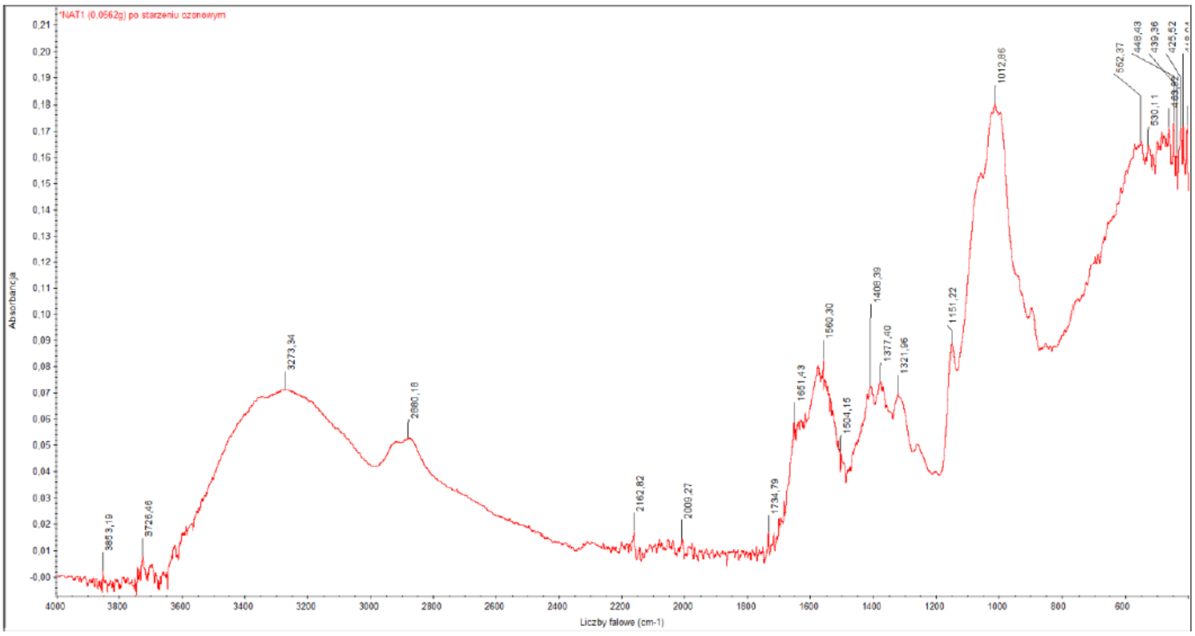

(b)

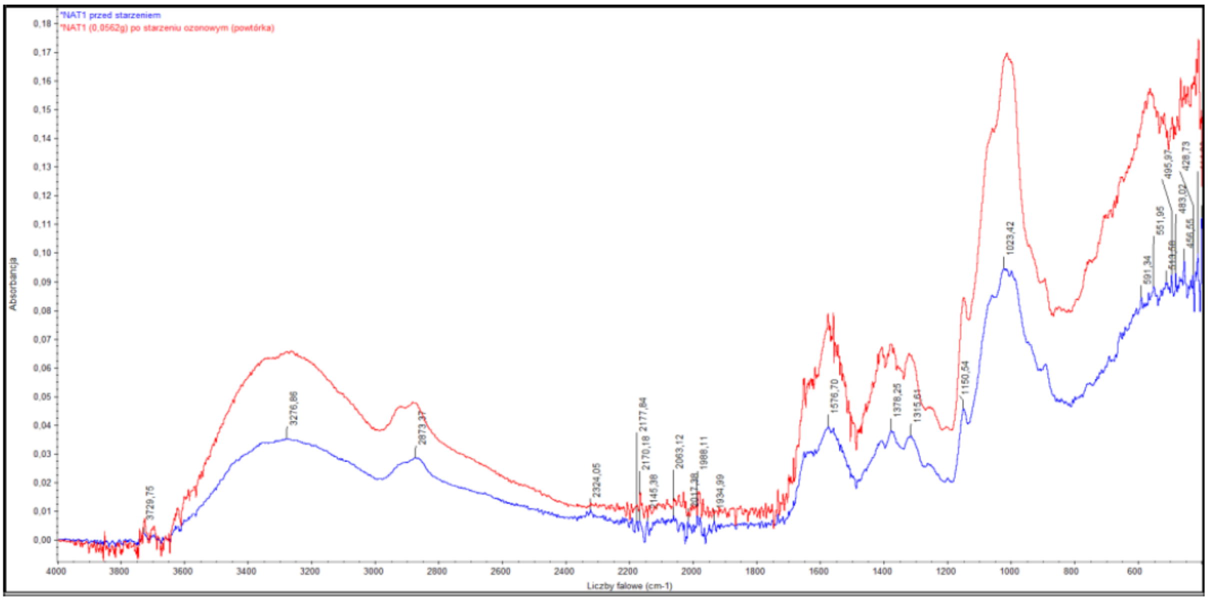

Figure S44. Summary of spectra before (a) and after (b) infrared ozone aging for CMS(1)/Ch(1)/Van(0,04).

(a)

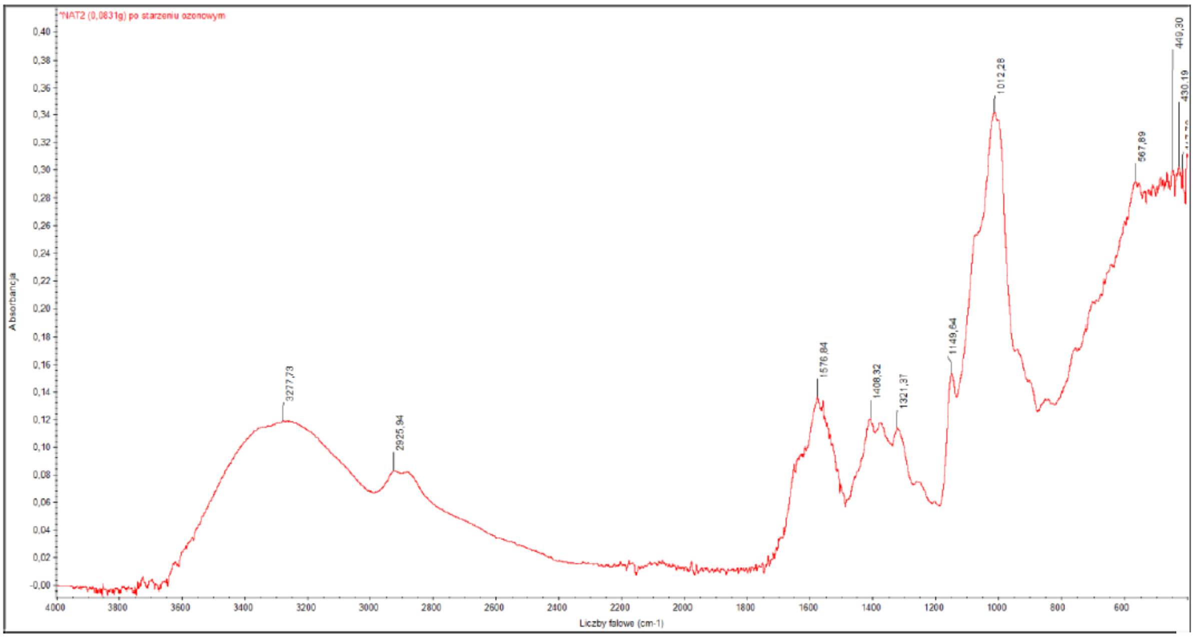

(b)

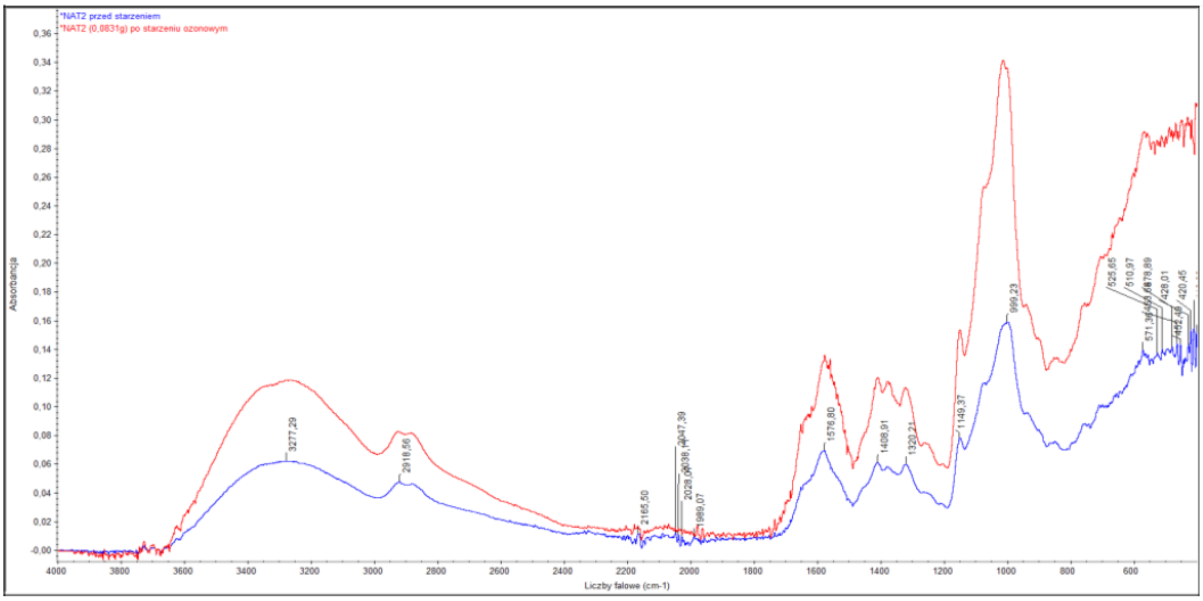

Figure S45. Summary of spectra before (a) and after (b) infrared ozone aging for CMS(1)/Ch(1)/Van(0,08).

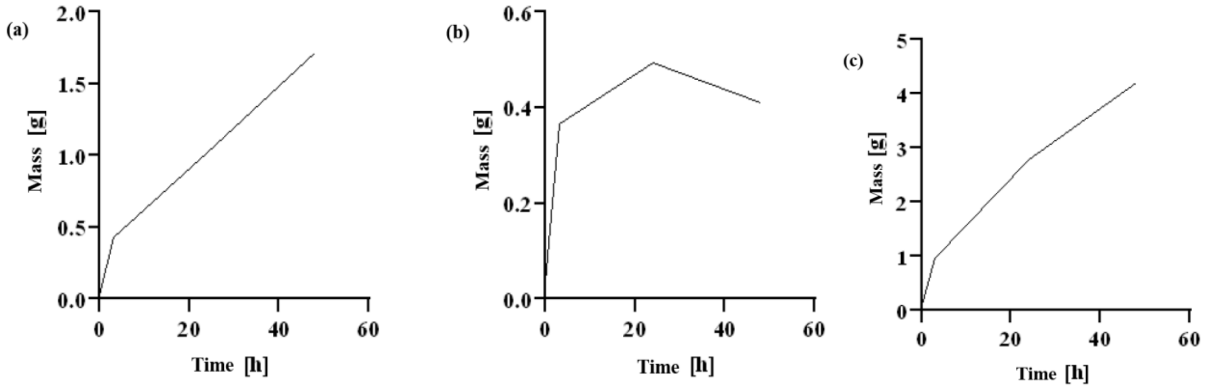

Figure S46. Time dependence of surface water absorption by the tested polymers: (a) CMS(1)/Ch(1)/Van(0,04); (b) CMS(1)/Ch(1)/Van(0,08); (c) CMS(1)/Ch(1).

Name sample	The process of preparing modular and seed material	Prepared composition	The process of germination on a given material
Native sample	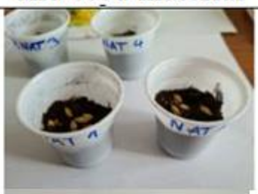	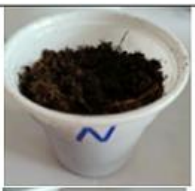	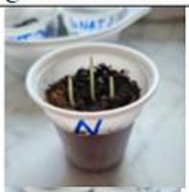
CMS(1)/Ch(1)/ Van(0.04)	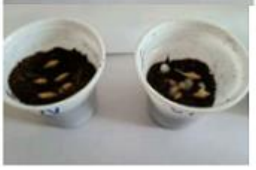	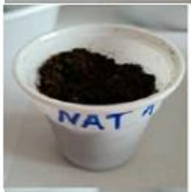	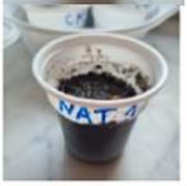
CMS(1)/Ch(1)/ Van(0.08)	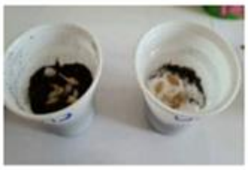	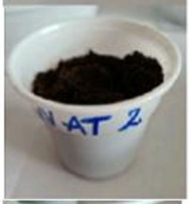	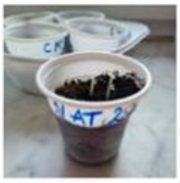
Chitosan	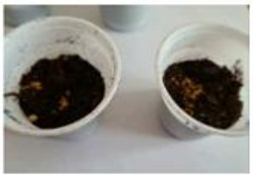	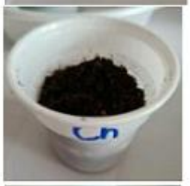	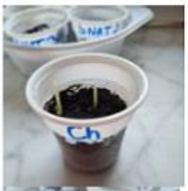
CMS	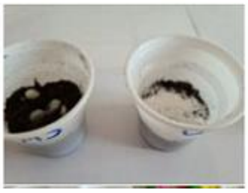	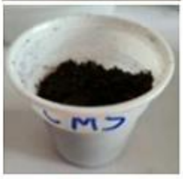	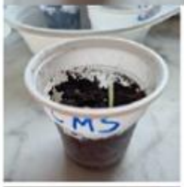
CMS(1)/Ch(1)	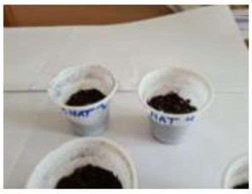	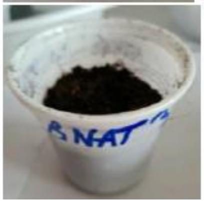	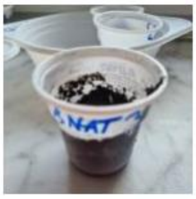

Figure S47. Sowing plants from the OECD group in containers with modular material and the germination process.

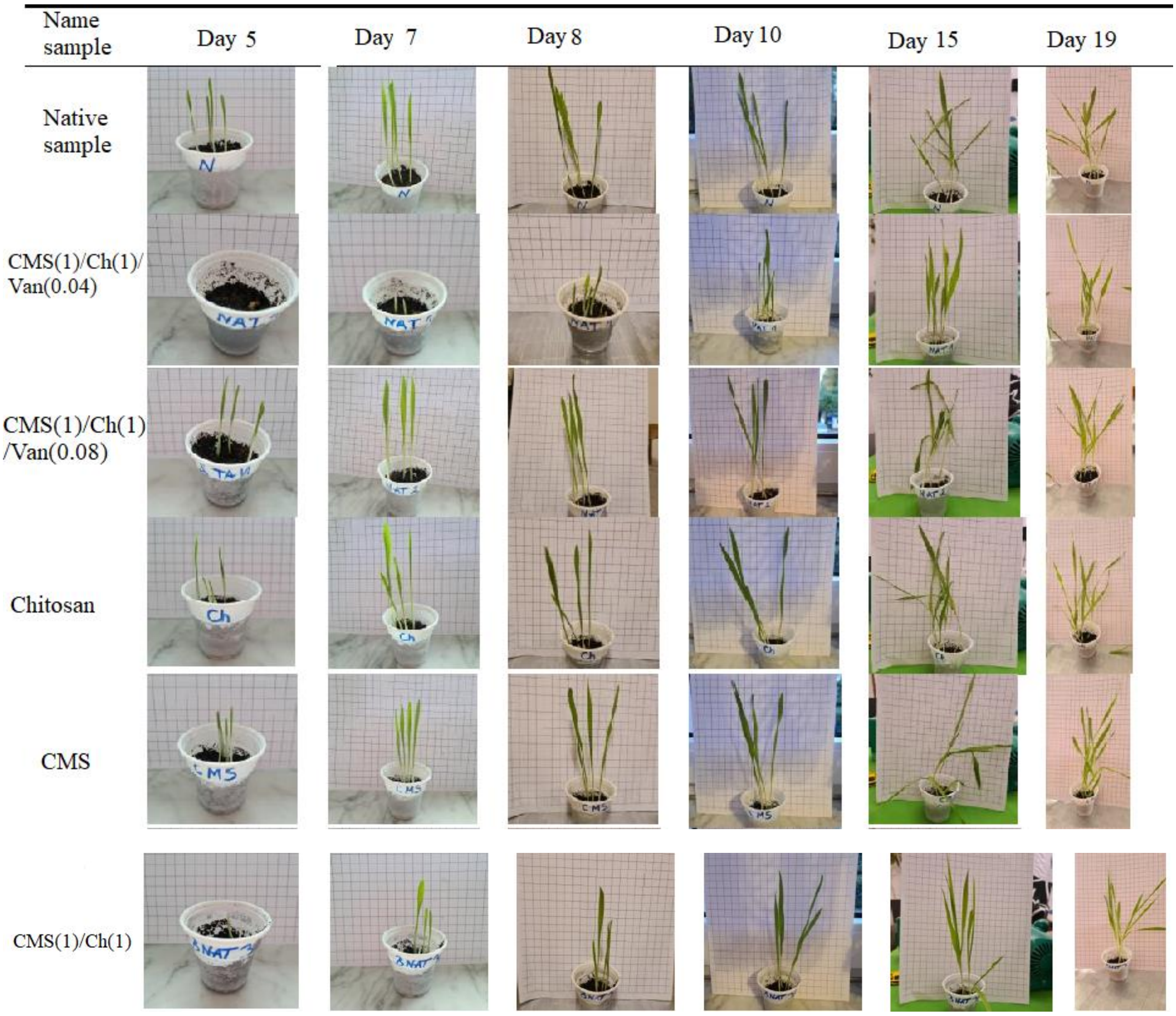

Figure S48. Further growth of OECD plants with tested polymers.

(a)

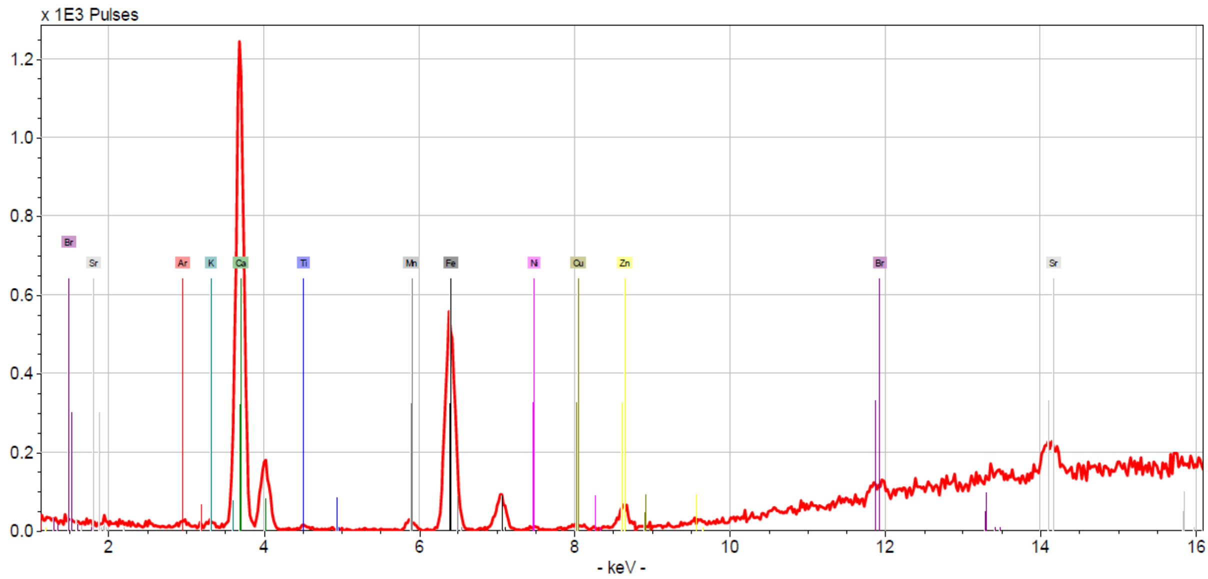
x 1E3 Pulses
1.2
1.0
0.8
0.6
0.4
0.2
0.0
Br
Sr
Ar
K
Ca
Ti
Mn
Fe
Ni
Cu
Zn
Br
Sr
2
4
6
8
10
12
14
16
- keV -

(b)

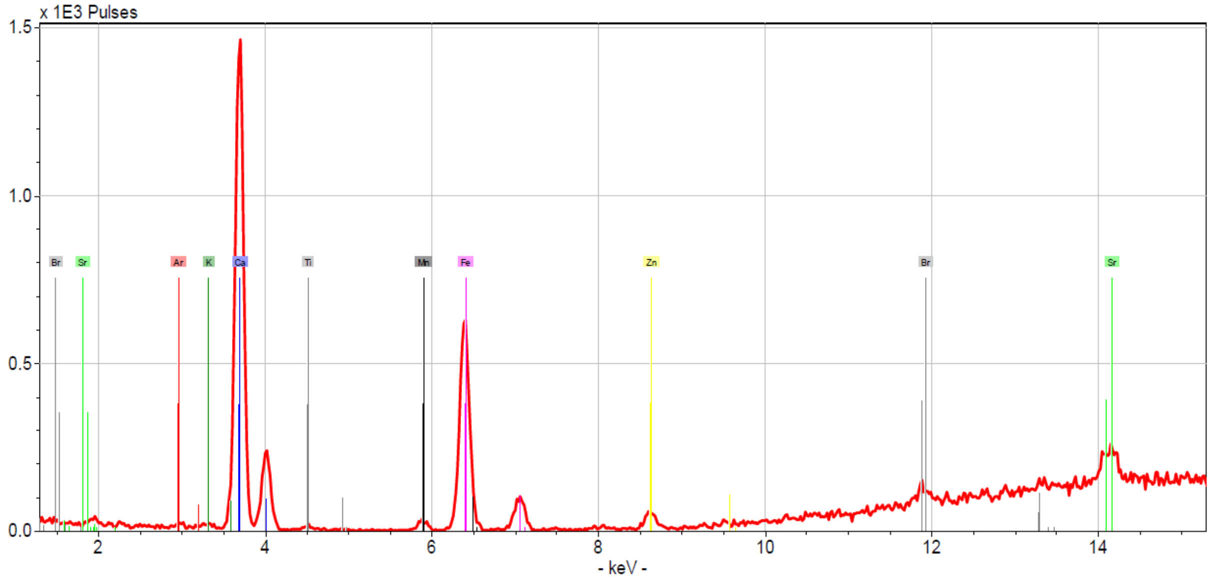
x 1E3 Pulses
1.5
1.0
0.5
0.0
Br
Sr
Ar
K
Ca
Ti
Mn
Fe
Zn
Br
Sr
2
4
6
8
10
12
14
- keV -

(c)

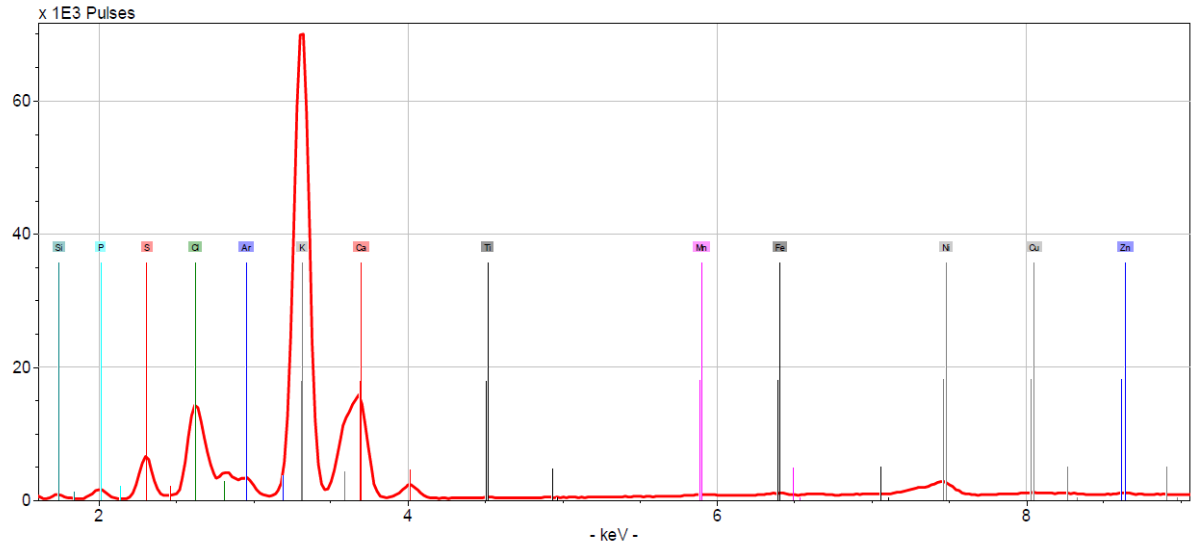

Figure S49. X-ray fluorescence spectroscopy (XRF) spectra for (a) CMS in soil; (b) CMS(1)/Ch(1)/Van (0.04) in soil; (c) CMS(1)/Ch(1)/Van(0.04) in straw.
